# Identification of putative effectors of the Type IV secretion system from the *Wolbachia* endosymbiont of *Brugia malayi*

**DOI:** 10.1371/journal.pone.0204736

**Published:** 2018-09-27

**Authors:** Emily M. Carpinone, Zhiru Li, Michael K. Mills, Clemence Foltz, Emma R. Brannon, Clotilde K. S. Carlow, Vincent J. Starai

**Affiliations:** 1 Department of Microbiology, University of Georgia, Athens, GA, United States of America; 2 Division of Genome Biology, New England Biolabs, Ipswich, MA, United States of America; 3 Department of Infectious Diseases, University of Georgia, Athens, GA, United States of America; Purdue University, UNITED STATES

## Abstract

*Wolbachia* is an unculturable, intracellular bacterium that persists within an extremely broad range of arthropod and parasitic nematode hosts, where it is transmitted maternally to offspring via vertical transmission. In the filarial nematode *Brugia malayi*, a causative agent of human lymphatic filariasis, *Wolbachia* is an endosymbiont, and its presence is essential for proper nematode development, survival, and pathogenesis. While the elucidation of *Wolbachia*:nematode interactions that promote the bacterium’s intracellular persistence is of great importance, research has been hampered due to the fact that *Wolbachia* cannot be cultured in the absence of host cells. The *Wolbachia* endosymbiont of *B*. *malayi* (*w*Bm) has an active Type IV secretion system (T4SS). Here, we have screened 47 putative T4SS effector proteins of *w*Bm for their ability to modulate growth or the cell biology of a typical eukaryotic cell, *Saccharomyces cerevisiae*. Five candidates strongly inhibited yeast growth upon expression, and 6 additional proteins showed toxicity in the presence of zinc and caffeine. Studies on the uptake of an endocytic vacuole-specific fluorescent marker, FM4-64, identified 4 proteins (*w*Bm0076 *w*Bm00114, *w*Bm0447 and *w*Bm0152) involved in vacuole membrane dynamics. The WAS(p)-family protein, *w*Bm0076, was found to colocalize with yeast cortical actin patches and disrupted actin cytoskeleton dynamics upon expression. Deletion of the Arp2/3-activating protein, Abp1p, provided resistance to *w*Bm0076 expression, suggesting a role for *w*Bm0076 in regulating eukaryotic actin dynamics and cortical actin patch formation. Furthermore, *w*Bm0152 was found to strongly disrupt endosome:vacuole cargo trafficking in yeast. This study provides molecular insight into the potential role of the T4SS in the *Wolbachia* endosymbiont:nematode relationship.

## Introduction

After leprosy, lymphatic filariasis is the leading cause of permanent disability, afflicting at least 150 million people worldwide [1, 2]. The World Health Organization reports that 1.23 billion people in 58 countries are at risk for developing the disease [1]. Lymphatic filariasis results from the mosquito-borne transmission of three distinct pathogenic nematodes: *Wuchereria bancrofti*, *Brugia timori* and *Brugia malayi*. In the early stages of the infection, anti-parasitic agents such as diethylcarbamazine and ivermectin have proved to be effective in eliminating immature worms and have been used in mass treatment programs [3]. Unfortunately, adult worms which are largely responsible for the pathology associated with infection cannot be easily treated by early-stage anti-parasitic drugs [4, 5]. With the discovery that these filarial nematodes require the presence of an obligate intracellular, Gram-negative bacterial endosymbiont of the *Wolbachia* genus to survive and reproduce, effective clearance of filarial infections has been achieved with antibiotic treatment [4–8]. Accordingly, significant efforts to understand the interactions of *Wolbachia* with nematodes at the molecular level have been undertaken to identify additional potential drug targets [9].

In *B*. *malayi*, *Wolbachia* exists and replicates within membrane-bound vacuoles in the lateral hypodermal chord and female germline [10–12]. These bacteria-laden compartments are often seen in close association with the endoplasmic reticulum and Golgi compartments [13], suggesting that *Wolbachia* may directly interact with host organelles to support its survival and co-opt normal host pathways in order to obtain nutrients and to prevent autophagic degradation in the host [14–16]. Only recently, however, have researchers identified a small number of secreted proteins from *Wolbachia* that appear to directly interact with host nematode proteins [17, 18]. Identification and characterization of the full complement of *Wolbachia*:host interactions will likely provide important information regarding the ability of *Wolbachia*, and other endosymbionts, to be maintained in the host.

Many Gram-negative intracellular bacteria utilize type III or type IV secretion systems (T3SS; T4SS) to deliver bacterial proteins into host cells, ensuring their successful invasion, survival, and replication within the host [19, 20]. Recent genomic studies have shown that *Wolbachia* from the filarial nematode *B*. *malayi* (*w*Bm) contain the necessary gene products to produce an active T4SS which is regulated by the *w*BmxR1 and *w*BmxR2 transcriptional regulators [16]. It is thought that *Wolbachia* utilizes its T4SS to secrete a number of effector proteins to modulate bacteria:host interactions, much like the related human pathogenic bacteria from the order Rickettsiales, such as *Rickettsia conorii*, *Ehrlichia chaffeensis*, and *Anaplasma phagocytophilium* [21]. However, identifying and characterizing these putative effectors has proven difficult in the filarial system due to the fact that the full life cycle of neither *Wolbachia* nor *B*. *malayi* can be supported in culture for facile genetic or biochemical manipulation. With modern bioinformatics, molecular biology, and a surrogate eukaryotic host system capable of genetic manipulation, the activities of individual putative effector proteins from these bacteria can be evaluated.

The budding yeast *Saccharomyces cerevisiae* has routinely been used as a “host” organism to screen for T3SS/T4SS effectors, as many of its core physiological pathways, such as protein trafficking, endolysosmal membrane dynamics, and cytoskeletal dynamics, are conserved in higher eukaryotes [22–24]. By expressing individual genes encoding predicted effector proteins in yeast, researchers have successfully identified and characterized several secreted effectors from a diverse set of bacterial pathogens including *Shigella flexneri* [25, 26], *Legionella pneumophila* [27, 28], and *Chlamydia trachomatis* [29]. Oftentimes, these effector proteins contain eukaryotic-like motifs, such as ankyrin repeat and coiled-coil domains–known protein-protein interaction motifs–suggesting the ability of these effectors to directly interact with and modulate eukaryotic cell biology. Secreted bacterial effectors with activities on eukaryotic physiology are often inhibitory to yeast growth [26] and can therefore be rapidly screened to identify potential candidates for further study. Additionally, powerful biochemical assays to measure protein trafficking pathways and the visualization of intracellular organelle morphologies can be used to identify effectors that may modulate intracellular membrane or general organelle dynamics during infection or symbiosis [30, 31].

By using some of these classical eukaryotic protein motifs as an identifier, and combining this information with genes known to be associated with, or co-transcribed with, the components of the *w*Bm T4SS [16, 32], we have identified and cloned forty-seven potential effector proteins from *w*Bm into yeast expression vectors. These putative effectors were then screened for the ability to inhibit yeast growth, alter normal vacuole protein sorting pathways, and to disrupt normal yeast vacuolar morphology. Five candidates strongly inhibit yeast growth upon expression, and six additional proteins show toxicity in the presence of zinc and caffeine. Studies on the uptake of an endocytic vacuole-specific fluorescent marker, FM4-64, identify 4 proteins (*w*Bm0076, *w*Bm00114, *w*Bm0152, and *w*Bm0447) capable of altering vacuole membrane dynamics. Additionally, we find that *w*Bm0076 disrupts normal actin cytoskeleton dynamics by inducing the aberrant formation of cortical actin patches *in vivo*. *w*Bm0152 expression strongly inhibits the delivery of representative endosomal protein cargo to the yeast vacuole, suggesting the ability of a *Wolbachia* protein to manipulate eukaryotic membrane traffic. These findings provide insight into the potential role of these proteins in the nematode:*Wolbachia* symbiotic relationship.

## Results

### Identification of a set of putative effectors from the *w*Bm genome

In a previous study [16], the existence of an active bacterial T4SS was confirmed in the *Wolbachia* endosymbiont of the filarial parasite *Brugia malayi*. Due to the probability that the T4SS acts as a major communication channel between *Wolbachia* and *B*. *malayi*, we set out to identify potential substrates/effectors of the *w*Bm T4SS. Using a variety of bioinformatic approaches (Materials and Methods), we identified 47 candidate effectors (Table 1) that displayed one or more of the following characteristics: **i**) eukaryotic-like protein possessing domains which share higher levels of homology to eukaryotic proteins than bacterial ones. These regions of homology include ankyrin repeats, Ser/Thr kinase motifs and coiled-coil domains, which have been previously identified in the T4SS effector proteins from *Legionella pneumophila* [33], *Bartonella henselae* [34], and *Coxiella burnetii* [35], **ii**) protein sequences that are potentially related to T4SS machineries, based on sequence homology to *Rickettsia sibirica* proteins shown to interact with components of its T4SS [36], or genes that are co-transcribed with components of the *wBm* T4SS [16], and **iii**) bacterial proteins identified in excretory-secretory products from *B*. *malayi* adult worms and microfilaria [37]. Each of the 47 open reading frames was cloned into the galactose-inducible yeast expression vector pYES2/NT A (see Materials and Methods), and further studied for their ability to modulate yeast physiology.

**Table 1:** *Wolbachia* putative Type IV effector proteins screened in this study.

*wBm* gene designation	Predicted function	Reason^a^	*wBm* gene designation	Predicted function	Reason^a^
***WBM0014***	**ABC transporter**	4	*WBM0430*	invasion associated protein B	S
***WBM0032***	**tRNA modification GTPase**	4	*WBM0432*	WSP family	S, *Bm*
*WBM0044*	amino acid transporter	*Bm*	*WBM0447*	ankyrin repeat protein	S, *Bm*, E
*WBM0057*	ZapA superfamily, hypothetical		*WBM0452*	SAM-dependent methyltransferase	E, 4
*WBM0064*	FAD-dependent oxidoreductase		*WBM0482*	hypothetical, tropomyosin-like	*Bm*, E
*WBM0070*	Mg^2+^/Co^2+^ transporter	4	***WBM0484***	**hypothetical protein**	S
***WBM0076***	**WAS(p) family protein, proline-rich**	E	*WBM0491*	cytochrome C oxidase assembly protein	S
***WBM0100***	***Wolbachia* Surface Protein (WSP) family**	S	*WBM0506*	outer membrane protein	E
***WBM0114***	**peptide deformylase**	4	*WBM0582*	ankyrin repeat protein	S
***WBM0152***	**PAL-like**	S, E	*WBM0665*	ankyrin repeat protein	4
***WBM0164***	**hypothetical, tropomyosin-like**	E	***WBM0666***	**dihydrolipoamide acetyltransferase**	4
***WBM0165***	**hypothetical protein**	U	*WBM0671*	DNA uptake lipoprotein	
***WBM0181***	**ATP-binding chaperone**	*Bm*, 4	***WBM0672***	**hypothetical protein**	*Bm*, U
***WBM0193***	**hypothetical, tropomyosin-like**	*Bm*, E	***WBM0709***	**coprophyrinogen III oxidase**	4
***WBM0209***	**pyruvate phosphate dikinase**	*Bm*	*WBM0711*	DNA recombination *rmuC*-like	S, *Bm*, 4
*WBM0213*	hypothetical protein	E, U	*WBM0736*	secreted FK506 binding protein-like	*Bm*
***WBM0222***	**preprotein translocase YajC**	4	***WBM0748***	**hypothetical protein**	4
***WBM0277***	**calcineurin-like phopshodiesterase**	4	*WBM0749*	hypothetical protein	*Bm*, 4
***WBM0284***	**WSP family**	4	*WBM0751*	hypothetical protein	4
*WBM0287*	ankyrin repeat protein	E	***WBM0752***	**hypothetical protein**	4
***WBM0290***	**D-alanyl D-alanine carboxypeptidase**	S	*WBM0772*	hypothetical protein	*Bm*, E
*WBM0307*	cytochrome C oxidase subunit	4	***WBM0791***	**N6-adenine methylase**	
***WBM0384***	**ankyrin repeat protein, metalloprotease**	E	***WBM0792***	**hypothetical protein**	E
*WBM0394*	ankyrin repeat protein	E			

^a^Characteristics of gene or protein product that supported selection: S, detected in *Wolbachia* secretome; *Bm*, detected in dissected *Brugia malayi*; E, predicted eukaryotic-like protein motifs; 4, co-regulated with *wBm* Type IV secretion apparatus or similar to predicted Type IV effectors from related organisms; U, unique to *Wolbachia*. ORFs noted in bold text were visualized to express in yeast via immunoblot (S1 Fig).

### Putative *w*Bm effectors inhibit yeast growth

**Fig 1:**
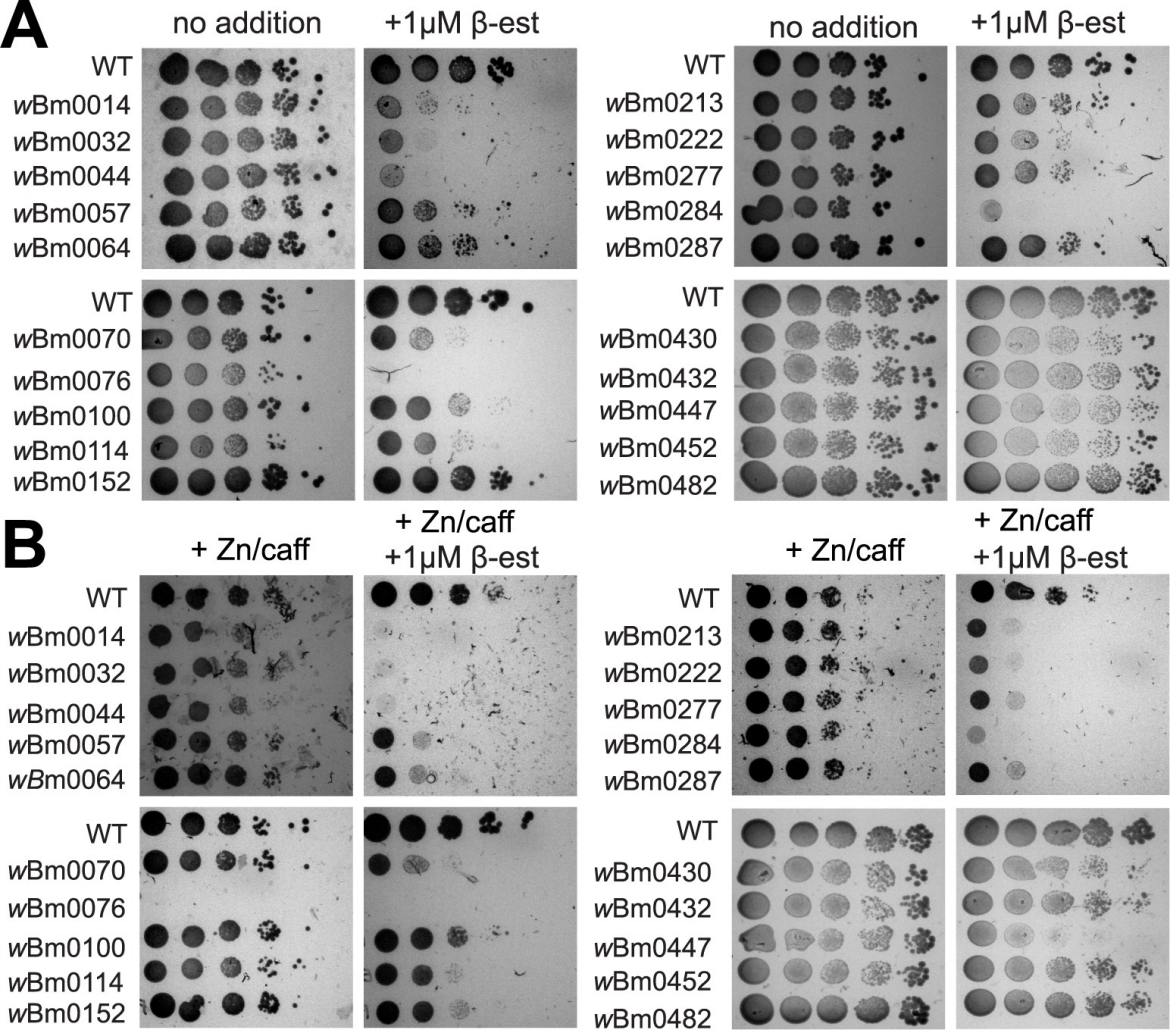
*w*Bm genes inhibit yeast growth upon expression. **(A)** BY4742 yeast strains genetically modified with GEV for β-estradiol-dependent induction of *GAL* promoters (Materials and Methods) and harboring the *GAL-*inducible control plasmid pYES2/NT A, or pYES2/NT A containing the specified *w*Bm open reading frame were grown to saturation in CSM-uracil medium containing 2% glucose, and each culture was diluted to OD_600_ = 1.0 in sterile 0.9% NaCl. 10-fold serial dilutions were spotted onto CSM-uracil containing 2% glucose with and without 1 μM β-estradiol. Plates were incubated for 48 h at 30°C. **(B)** Strains and dilutions from **(A)** were spotted to CSM-uracil media containing 2% glucose supplemented with 7.5 mM ZnCl_2_, 5 mM caffeine, and with or without 1 μM β-estradiol. Plates were incubated for 72 h at 30°C. Images are representative of three independent experiments.

In an effort to identify those *w*Bm proteins that manipulate eukaryotic physiology, we tested the ability of each of the putative effectors to inhibit yeast growth; many bacterial effector proteins have been identified in such a manner [25–29]. Each galactose-inducible plasmid harboring an individual *w*Bm “effector” was transformed into a yeast strain and gene expression was induced via a modified Gal4p transcriptional regulator which responds to the hormone β-estradiol, instead of the usual galactose (Materials and Methods), thus allowing *GAL* promoter induction with the same carbon source as uninduced conditions (glucose) and preventing any confounding results that may be introduced by changing carbon sources across expression conditions. Under growth conditions containing β-estradiol, we were able to detect full-length protein expression of 24 of the 47 putative effector proteins via the N-terminal epitope provided by the pYES2/NT A expression plasmid (S1 Fig, also noted on S1 Table). It is likely that the total number of expressed proteins is underrepresented using this detection method, however, since N-terminal protein processing or degradation events may occur upon protein overproduction *in vivo*; further possibilities that could result in translation defects of these putative effectors were not explored. When strains harboring these plasmids were grown on minimal medium with or without β-estradiol, we found *w*Bm0014, *w*Bm0032, *w*Bm0044, *w*Bm0076, and *w*Bm0284 strongly inhibit yeast growth in the presence of 1 μM β-estradiol (determined by a reduction in cell titers by three orders of magnitude), but not in the absence of inducer (Fig 1A). Slight inhibition in yeast growth (as determined by a reduction in cell titers by two orders of magnitude) was observed upon expression of *w*Bm0070, *w*Bm0114, and *w*Bm0222 (Fig 1A). Other expressed *w*Bm genes did not strongly inhibit the growth of yeast under these conditions (S2 Fig).

Due to the ability of *Wolbachia* to live inside membrane-bound compartments in *B*. *malayi* cells [13], we next hypothesized that *Wolbachia* could secrete proteins that subvert normal endolysosomal membrane dynamics, potentially as a method of avoiding autophagic degradation [38, 39]. As yeast strains are known to require normal endolysosomal or vacuolar function to resist high levels of zinc and caffeine [40, 41], we tested the ability of these *w*Bm genes to induce growth sensitivity to 7.5 mM ZnCl_2_ and 5 mM caffeine. As expected, putative effectors that were toxic on standard minimal media showed enhanced β-estradiol-dependent toxicity on zinc/caffeine plates (Fig 1B). Interestingly, strains harboring the *w*Bm0076 construct were sensitive to zinc/caffeine even under non-induction conditions (Fig 1B, lower left), suggesting that even low-level expression of *w*Bm0076 is sufficient to inhibit yeast growth under these conditions. Because expression of each of the eight above identified putative effectors alone is sufficient to inhibit yeast growth under normal growth conditions, no conclusions can be drawn from the similarly defective growth observed in the presence of both β-estradiol and zinc/caffeine; it is not known if this observed toxicity is a result of protein-induced endolysomal membrane trafficking defects or other protein-induced defects to yeast biology. Of note, however, was the discovery that *w*Bm0057, *w*Bm0064, *w*Bm0213, *w*Bm0277, *w*Bm0287 and *w*Bm0447 were only strongly toxic in the presence of zinc and caffeine (as determined by an observable reduction in cell titers by three orders of magnitude) (Fig 1B), suggesting that these strains may have defects in normal endolysosomal homeostasis, such as in defects in the TOR kinase pathway [42, 43] or protein trafficking pathways [44], although defects in DNA repair pathways may also contribute to caffeine sensitivity [45]. No other plasmids were found to induce additional sensitivity of yeast to zinc/caffeine treatment upon expression, though several others show slight inhibition of yeast growth (S2 Fig). Taken together, we find several putative effector proteins from *Wolbachia* that inhibit the growth of yeast, suggesting these proteins may function to modulate essential eukaryotic pathways during *Wolbachia* symbiosis.

### Putative *Wolbachia* effectors disrupt yeast vacuole membrane dynamics

As reported previously, *Wolbachia* likely avoids both lysosomal and autophagic degradation pathways to survive within a membrane-bound organelle since chemical activation of autophagy results in clearance of *Wolbachia* from maturing nematodes [39]. Due to the fact that *w*Bm must avoid normal host autophagic pathways, it is likely that *w*Bm secretes proteins that alter normal host endolysosomal membrane trafficking pathways. The degradative vacuole of yeast has long been used as a model organelle for mammalian lysosome function and eukaryotic endolysosomal fusion dynamics [46], therefore we assayed the effects of *w*Bm putative effector expression on yeast vacuole dynamics.

The yeast vacuole is a highly dynamic organelle that undergoes constant rounds of fission and fusion in response to the osmolarity of the extracellular environment [47]. The homotypic fusion of yeast vacuoles, like the majority of eukaryotic intracellular membrane fusion events, is governed by a conserved set of protein machinery consisting of soluble NSF attachment protein receptors (SNAREs), a Rab-family small GTPase, and a Rab/SNARE-binding multisubunit tethering factor, the homotypic vacuole fusion and protein sorting (HOPS) complex (reviewed in [48]). Yeast strains defective in the proper regulation of homotypic fusion, either directly or indirectly, generally display aberrant vacuolar morphologies. These phenotypic changes have long been used to identify important genes and regulators of endolysosomal membrane fusion and protein trafficking machineries in yeast [41, 49]. Six different classes of vacuoles have been described, classified as A through F [50]; class A vacuoles are considered a wild type morphology. The remaining classes B-F are considered abnormal vacuole morphologies and are phenotypically described briefly: class B vacuoles represent highly fragmented vacuoles containing more than twenty vacuoles per cell, class C vacuoles are highly fragmented with no discernable vacuolar structure, class D vacuoles have a single enlarged vacuole, class E vacuoles have an enlarged prevacuolar compartment with small vesicles present, and class F vacuoles have many small vacuoles surrounding a larger prominent vacuole. Importantly, the expression of some secreted effectors from pathogenic bacteria in yeast show the ability to both fragment the yeast vacuole *in vivo* and inhibit the fusion of yeast vacuoles *in vitro* [40, 51, 52].

**Fig 2:**
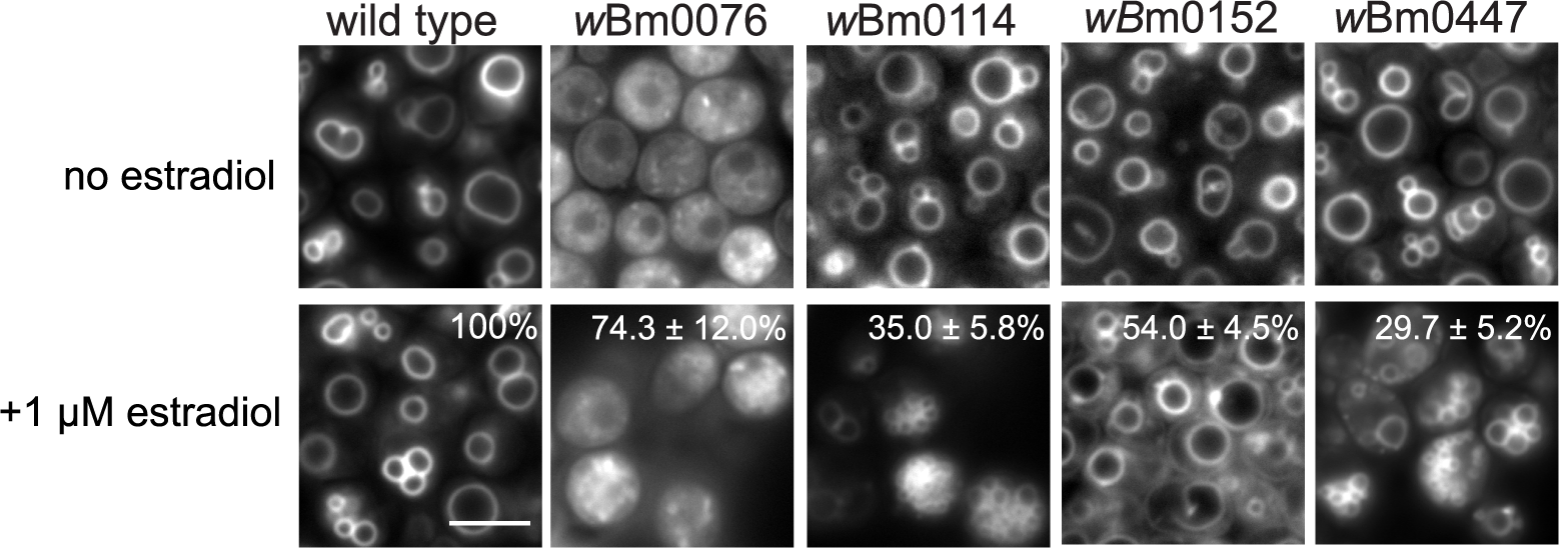
Normal vacuole dynamics are altered upon *wBm* gene expression. BY4742 yeast strains modified with GEV for β-estradiol-dependent induction of *GAL* promoters (Materials and Methods), and harboring *GAL*-inducible pYES2/NT A control plasmid or pYES2/NT A containing an individual *w*Bm open reading frame were grown to saturation in CSM-uracil medium, subcultured to CSM-uracil and grown for 6h at 30°C with or without the addition of 1 μM β-estradiol. Cells were stained for 20 minutes with 10 μM FM4-64 at 30°C followed by a 1.5 h chase in CSM-uracil at 30°C. Vacuoles were visualized via microscopy, and cells displaying abnormal vacuole morphologies were counted. Percentage and standard deviation of cells displaying the corresponding abnormal vacuolar morphology is noted and determined from three independent experiments; *n* ≥ 100 cells per experiment. Bar = 3 μ.

Yeast strains harboring a control plasmid under induction conditions (1 μM β-estradiol) show 1–5 large vacuoles per cell, as stained by the endocytic uptake of the vacuole-specific fluorescent marker, FM4-64 [30] (Fig 2). Under the same growth conditions, strains expressing *w*Bm0076 display an apparent highly fragmented, class C vacuole morphology in approximately 74% of the cells observed, often indicative of either a complete block in vacuole biogenesis, including defects in homotypic vacuole fusion, or disruptions in the delivery of FM4-64-containing endocytic vesicles to the vacuole [53, 54]. In strains expressing *w*Bm0114, approximately 35% of the observed cells display a large number of small, vacuole-like, class B compartments that may indicate defects in vacuolar protein sorting pathways [53, 55]. In the presence of *w*Bm0152, more than half of the yeast cells contain large vacuolar compartments with accumulations of small membrane compartments immediately adjacent to the vacuolar limiting membrane; these accumulations are likely enlarged pre-vacuolar compartments (class E compartments) that arise in strains defective in endosome:vacuole fusion or endosomal sorting complexes required for transport (ESCRT) function [50, 56]. Finally, approximately 30% of cells expressing *w*Bm0447 display an aberrant vacuole morphology that was not easily classified: most cells were multi-vacuolar with small membrane accumulations at the plasma membrane (Fig 2). No other strains harboring *w*Bm genes displayed a significant defect in yeast vacuole morphology (> 85% wild type vacuoles, S3 Fig); the ability of the selected *w*Bm proteins to strongly manipulate yeast endolysosomal membrane dynamics is specific to a select few putative effectors.

### *w*Bm0152 disrupts endosome:Vacuole cargo trafficking in yeast

**Fig 3:**
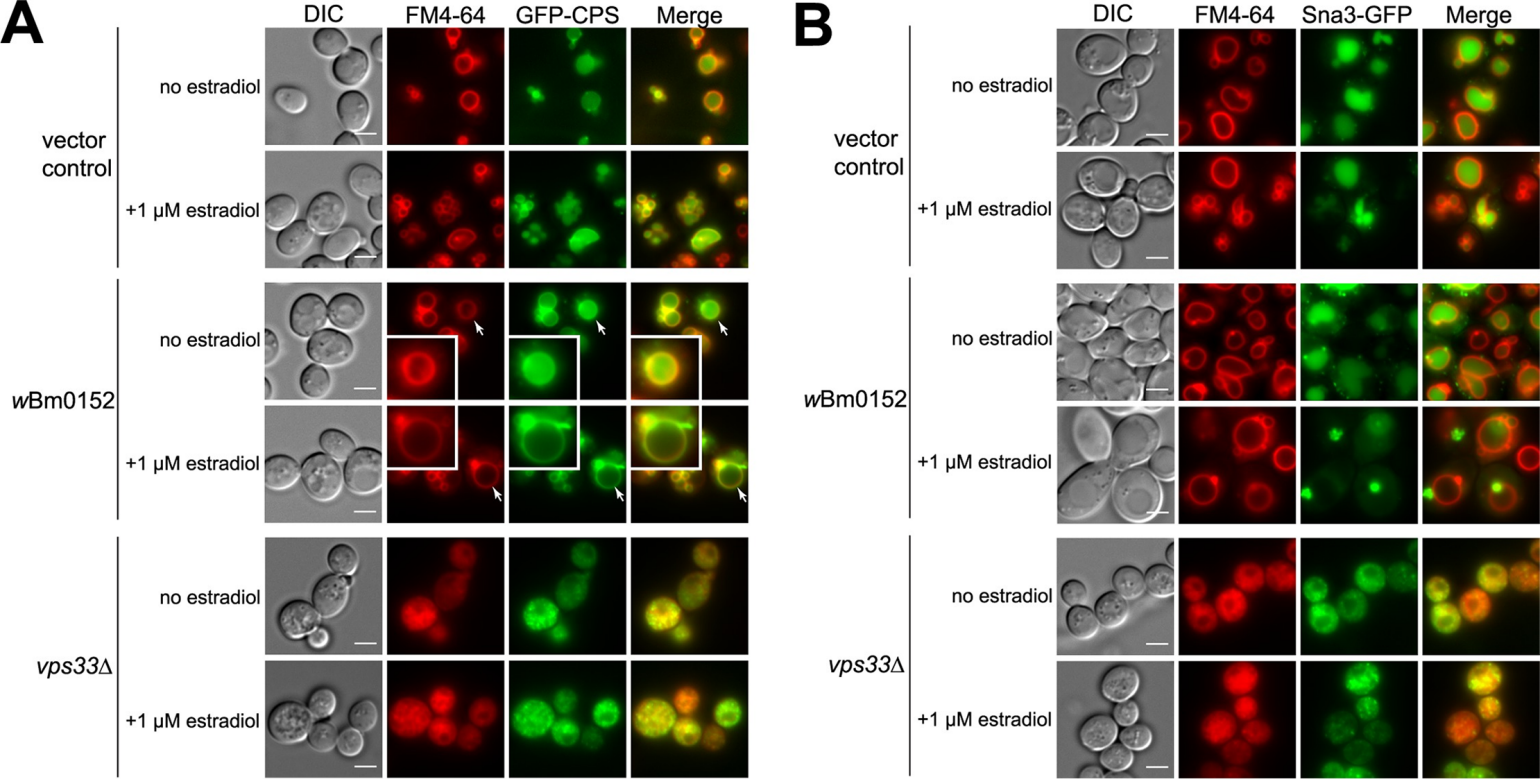
wBm0152 expression inhibits endosomal traffic to the yeast vacuole. BY4742 yeast strains modified with GEV for β-estradiol-dependent induction of *GAL* promoters (Materials and Methods), and harboring **(A)** GFP-CPS plasmid or **(B)** Sna3-GFP plasmid in addition to pYES2/NT A control plasmid or pYES2/NT A *w*Bm0152 were grown to saturation in CSM-lysine-uracil medium. Cells were subcultured to CSM-lysine-uracil with and without the addition of 1 μM β-estradiol and grown for 6h at 30°C. Cells were stained with 10 μM FM4-64 dye for 20 minutes at 30°C and chased 1.5h in CSM-lysine-uracil medium at 30°C. Cells were visualized, and representative images are shown. Bar = 3 μ. Inset in **(A)** magnifies the cell denoted by the arrow to better visualize the GFP-CPS trafficking defect in cells expressing *w*Bm0152; images are representative of two independent experiments.

To further characterize the potential defects in endolysosomal trafficking caused by the expression of putative *w*Bm effector proteins, we decided to identify *wBm* proteins that caused aberrant trafficking of endosome:vacuole cargo upon expression. We employed both GFP-tagged carboxypeptidase S (CPS), a membrane-bound vacuolar protease [57], and GFP-tagged Sna3p, a transmembrane ubiquitin ligase adapter protein involved in vacuole protein sorting [58]. It is known that both proteins cargo traffic to the yeast vacuole via an endosomal/multivesicular body (MVB) intermediate compartment [31, 57]. Under normal growth conditions of wild type strains, both GFP-CPS and Sna3-GFP localize to the yeast vacuole regardless of the presence of β-estradiol (Fig 3A and 3B). *VPS33* encodes for the Sec1/Munc18-family protein, which is a member of the multisubunit class C core vacuole/endosome tethering (CORVET) complex required for endosome:endosome fusion events. It is also a member of the multisubunit homotypic fusion and vacuole protein sorting (HOPS) complex required for both homotypic vacuole and late endosome:vacuole fusion [59–62]. In the strain lacking VPS33, both GFP-CPS and Sna3-GFP were found in aberrant intracellular compartments, as expected (Fig 3A and 3B, *vps33Δ*).

Upon induction with β-estradiol, most putative *wB*m effector proteins did not alter the normal vacuolar localization of either GFP-CPS (S4 Fig) or Sna3-GFP (S5 Fig), suggesting that expression of these proteins does not strongly alter normal endosome:vacuole traffic in yeast. Expression of the predicted peptidoglycan associated lipoprotein (PAL) *w*Bm*0152*, however, prevented both GFP-CPS and Sna3-GFP from accumulating within the vacuole lumen (Fig 3A and 3B, respectively), suggesting that this protein is capable of modulating the MVB:vacuole fusion event, and may play some role in either preventing the degradation of *Wolbachia* in nematode lysosomes, or supporting the synthesis of the *Wolbachia*-containing vacuole from post-Golgi membranes.

### Expression of *w*Bm0076 disrupts the yeast actin cytoskeleton

**Fig 4:**
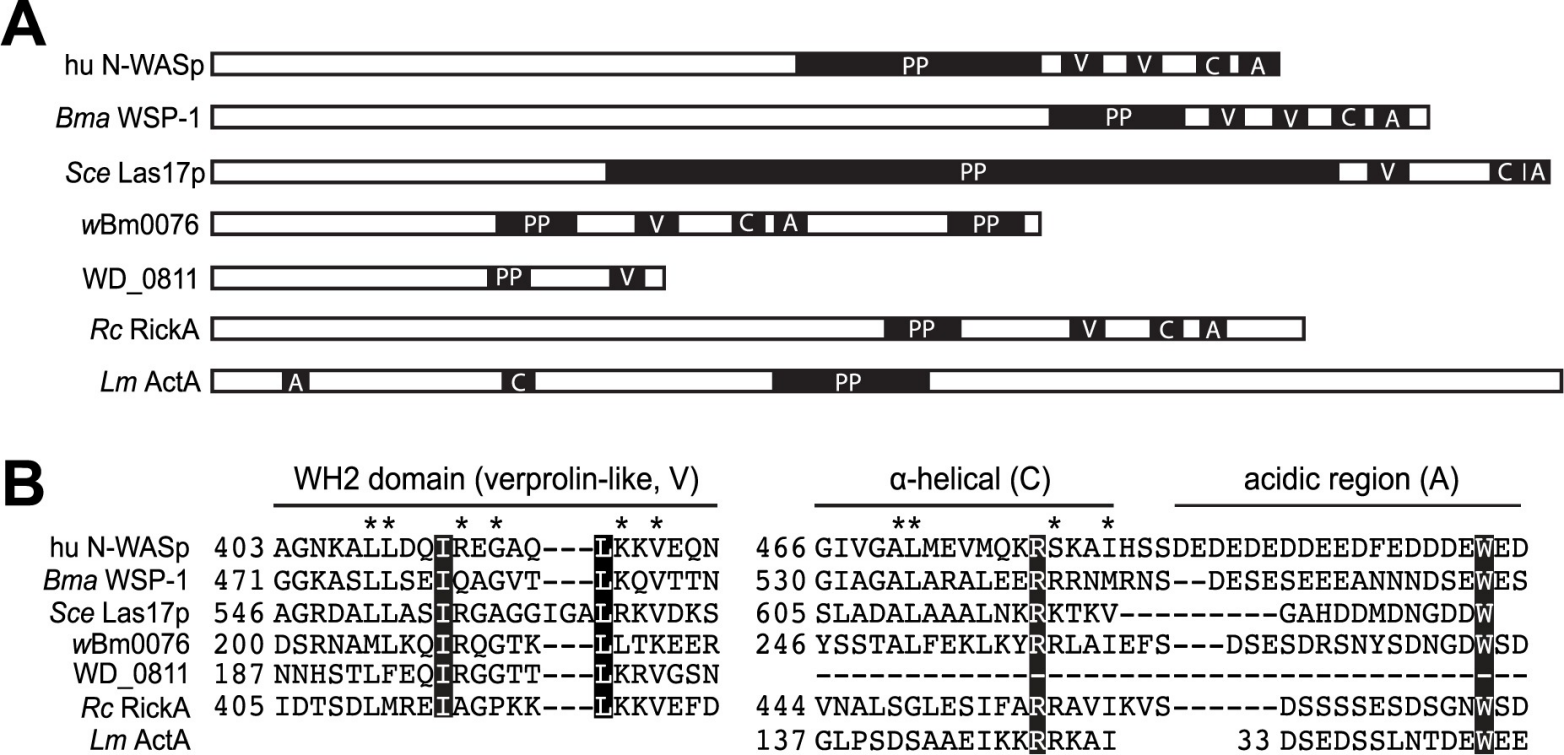
*w*Bm0076 is a WAS(p)-family protein. Domain structure of *w*Bm0076 orthologs, including: human N-WASp (hu N-WASp; BAA20128.1), *Brugia malayi* WSP-1 (*Bma* WSP-1; CRZ22528.1), *S*. *cerevisiae* Las17p (*Sce* Las17p, NP_014824.1), *w*Bm0076 (WP_011256278.1), *w*Mel WD_0811 (WD_0811; AAS14498), *Rickettsia conorii* RickA (*Rc* RickA; WP_041471735.1), and *Listeria monocygenes* ActA (*Lm* ActA; ABC40914.2). Domains including poly-proline motifs (PP), the WH2/verprolin-like (V), central α-helical (C), and acidic regions (A) are depicted. **(B)** Sequence alignment of the conserved VCA domains of the proteins in **(A)**. Completely conserved residues are highlighted with a black box and residues conserved in more than half of the sequences are denoted with an asterisk. Only the first WH2 domains of hu N-WASP and *Bma* WSP-1 are aligned for simplicity.

Expression of the *w*Bm0076 gene is lethal to yeast (Fig 1). Analysis of the protein sequence via BLAST searching identifies orthologs in closely related bacteria, including: *Wolbachia* of *Onchocerca volvulus* (WP_025264345.1), *Wolbachia* of *Onchocerca ochengi* (WP_014868845.1), *Wolbachia* of *Drosophila melanogaster* (AAS14498), and RickA from *Rickettsia conorii* (WP_041471735.1). Wbm0076 is a protein of 392 amino acids in length, containing a putative WH2 (verprolin, V) domain, an ~18 amino acid actin-binding motif, a central α-helical domain (C), an acidic region (A), and 2 proline-rich domains with homology to proteins of the eukaryotic neural Wiskott-Aldrich syndrome protein (N-WASP/WAS) family [63], including the bacterial RickA and actin-polymerizing ActA from *Listeria monocytogenes* [64] (Fig 4A and 4B). N-WASP proteins are known to bind actin monomers and the conserved eukaryotic Arp2/3 complex to stimulate branched actin filament formation in an Arp2/3-dependent manner [65, 66]. In addition, the RickA protein was determined to polymerize actin ‘comet tails’ on the surface of *Rickettsia* to aid in intracellular motility during infection, and to polymerize branched actin structures in a Arp2/3-dependent manner *in vitro* [67]. Based on the homology of *w*Bm0076 protein to both eukaryotic and bacterial proteins known to modulate actin structures, we hypothesized that *w*Bm0076 was likely modifying actin structures in yeast upon expression.

**Fig 5:**
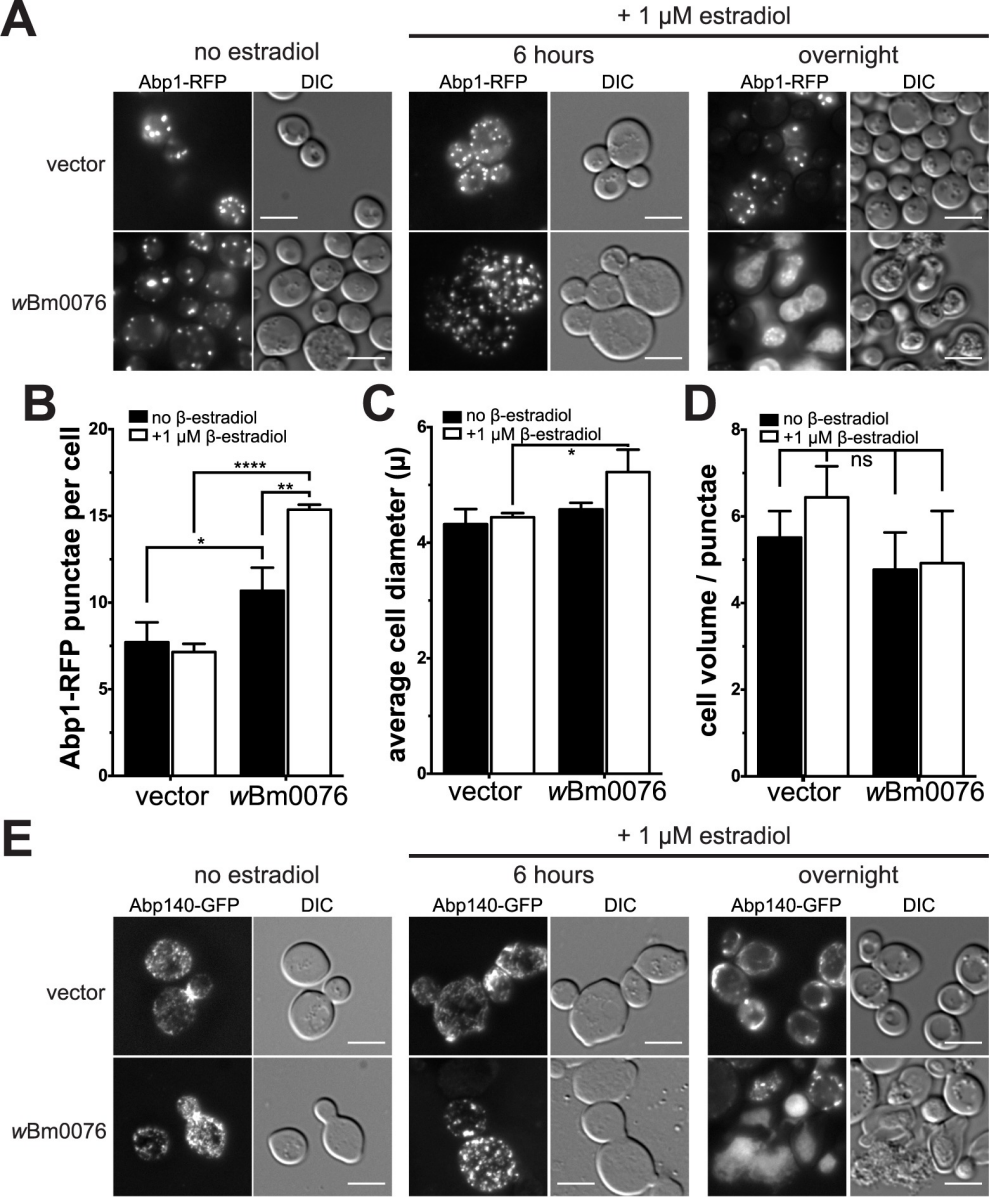
*w*Bm0076 expression alters yeast actin dynamics in vivo. S288C yeast strains modified with GEV for β-estradiol-dependent induction of *GAL* promoters (Materials and Methods), expressing **(A)** Abp1-RFP and harboring either the pYES2/NT A control plasmid or pYES2/NT A *w*Bm0076 were grown to saturation in CSM-uracil medium, subcultured to CSM-uracil, with or without 1 μM β-estradiol, for 6h at 30°C. Cells were harvested at indicated time points and visualized. Bar = 5 μ. **(B-D)** The average ± standard deviation Abp1-containing punctae are plotted per cell **(B)**, cell diameter **(C)**, and estimated cell volume / punctae **(D**, assume cell is a sphere during volume calculation) were measured from cells grown in **(A)**, and ≥100 cells each per three independent experiments were measured; ns = P > 0.05 (not significant); (*) = P ≤ 0.05; (**) = P ≤ 0.01; (****) = P ≤ 0.0001. **(E)** As in **(A)**, except yeast cells harbored Abp140-3xGFP instead of Abp1-RFP.

The yeast actin cytoskeleton consists of two main conformations: tight bundles of many actin filaments (cables) [68], and concentrated patches of highly branched actin that accumulate near the periphery of the yeast cell; these cortical actin patches are linked to newly-formed endocytic vesicles, thus enabling their retrograde trafficking [69]. To visualize cortical actin patches in the presence of *w*Bm0076, we used a yeast strain expressing RFP-tagged Abp1p. Abp1p is an actin filament binding protein which interacts with Arp2/3 to stimulate Arp2/3 activity [70], and regulates the capping of barbed-end actin filaments when bound to Aim3p [71]. In a yeast strain harboring a vector control, Abp1-RFP localizes to several cortical punctae per cell, as expected (Fig 5A). The presence of the *wBm0076* plasmid under non-inducing conditions does not alter the punctate localization of Abp1-RFP (Fig 5A). Upon addition of 1 μM β-estradiol, however, yeast expressing *w*Bm0076 display an increase in both in the number of Abp1-RFP punctae and cell size after 6 hours, when compared to the vector control strain (Fig 5A and 5B). While the overall number of cortical actin patches in *w*Bm0076-expressing strains did not appear to increase when correcting for the estimated cell volume (Fig 5B, right panel), it is not yet clear if the increased number of cortical actin patches increased the cell volume, or if the increased cell volume induced a corresponding increase in cortical actin patches. When left in inducing conditions overnight, however, Abp1-RFP-containing punctae in the *wBm0076*^*+*^ strain are no longer detected and cells appear to lyse through a local disruption in the cell wall.

To visualize actin cables *in vivo*, we used GFP-tagged Abp140p, the actin-binding tRNA methyltransferase responsible for the addition of 3-methylcytidine in the anticodon loop of yeast tRNAs [72]. The N-terminal actin-binding domain of Abp140p is known to localize to both actin cable and cortical actin patches; control yeast expressing Abp140-3xGFP clearly mark actin cables, with a higher local concentration around the bud neck, as expected (Fig 5C). Upon a 6-hour induction with 1 μM β-estradiol, cells harboring the *w*Bm0076 plasmid no longer show elongated actin cables, but rather a high concentration of discrete punctae similar to those seen in Abp1-RFP cells (Fig 5C); extended incubations in β-estradiol confirm the ability of *w*Bm0076 to lyse yeast cells (Fig 5C). Taken together, expression of *w*Bm0076 in yeast leads to an extensive disruption of the yeast actin cable network, an increase in the number of cortical patches containing branched actin, and cell lysis.

### Deletion of the Arp2/3-activating protein ABP1 provides resistance to *w*Bm0076 expression

The isolation of yeast mutant derivatives that are no longer sensitive to *w*Bm0076 expression could provide insight into not only the cellular processes modulated by *w*Bm0076, but also help elucidate the protein targets or biochemical activity of *w*Bm0076. Because *w*Bm0076 protein displays homology to WAS(p)-family proteins and disrupts the yeast actin cytoskeleton *in vivo*, we hypothesized that deletions of genes encoding for proteins known to be involved in regulating actin dynamics may provide some resistance to *w*Bm0076 expression in yeast.

**Fig 6:**
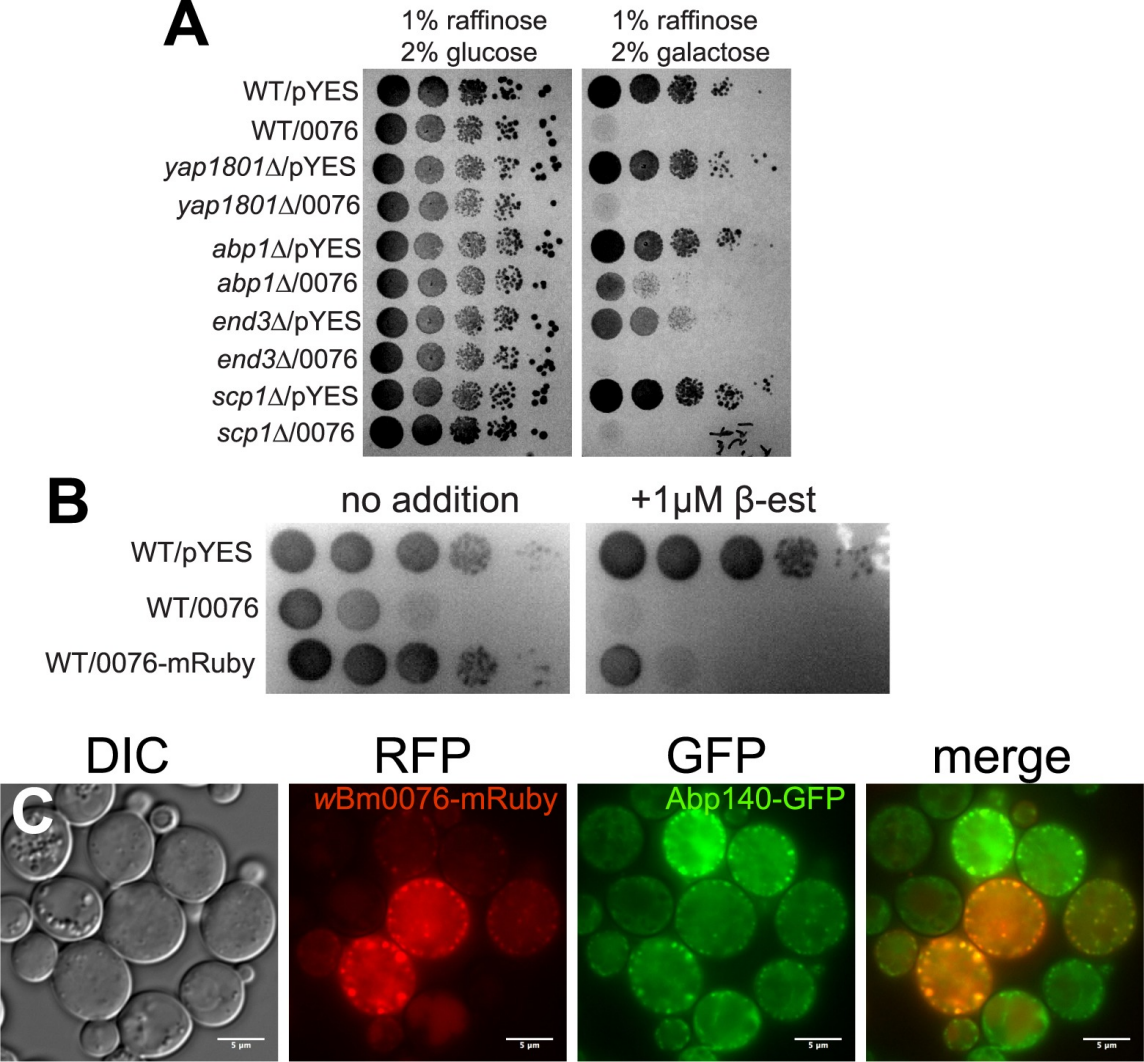
Deletion of *ABP1* reduces the toxicity of *w*Bm0076 expression. **(A)** BY4742 yeast strains deleted for the indicated gene and harboring either pYES2/NT A or pYES2/NT A *w*Bm0076 (0076) were grown overnight in CSM medium lacking uracil. Cultures were diluted to an OD_600_ = 1.0 in sterile 0.9% NaCl, then spotted in 10-fold dilutions on plates containing 1% raffinose and either 2% glucose or 2% galactose to induce *w*Bm0076 expression. Plates were incubated for 72 h at 30°C and imaged. **(B)** BY4742 yeast strains modified with GEV for β-estradiol-dependent induction of *GAL* promoters and harboring either pYES2/NT A (pYES), pYES2/NT A *w*Bm0076 (0076), or pYES2/NT A *w*Bm0076-mRuby2 (0076-mRuby) were grown spotted as in **(A)**, except that plates either contained or lacked 1 μM β-estradiol to induce *w*Bm0076 expression. Plates were incubated for 48 h and imaged. **(C)** Yeast strains harboring Abp140-3xGFP and pYES2/NT A *w*Bm0076-mRuby2 were grown overnight in CSM medium lacking uracil and subcultured to fresh CSM-Ura containing 1 μM β-estradiol. After 5 h outgrowth at 30°C, cells were harvested and visualized. Images are representative of three independent experiments; bar = 5μ.

In yeast, Las17p is the sole WAS(p)-family protein present that interacts with a large number of proteins via its conserved WH1 and proline rich domains and plays an important role in regulating endocytosis [73–76]. We therefore assayed the toxicity of *w*Bm0076 expression in thirty-one single gene deletion backgrounds, each of which were selected by their ability to produce proteins that either directly interact with Las17p, or otherwise regulate actin dynamics. Deletion strains harboring *w*Bm0076 or vector only controls were serially diluted and grown on minimal media containing 1% raffinose plus either 2% glucose (uninduced condition) or 2% galactose (induced). As expected, wBm0076 was toxic to yeast upon galactose induction (Fig 6A; WT/0076). We noted that some of these deletion strains (*arf1Δ*, *sho1Δ*, *vrp1Δ*, *las17Δ*, *bbc1Δ*, *myo5Δ*) grew very poorly on media containing galactose (S6 Fig), even in the absence of *w*Bm0076, and we therefore could not easily interpret these results. Importantly, expression of *w*Bm0076 did not restore the ability of the *las17Δ* strain to grow on galactose (S6 Fig); it is therefore unlikely that *w*Bm0076 directly complements Las17p activity in *Saccharomyces*. However, of the thirty-one deletion strains tested, only *abp1Δ* strains displayed resistance to expression of *w*Bm0076 on media containing galactose (Figs 6A and S6; *abp1Δ/*0076).

Because Abp1p localizes to cortical actin patches and its deletion limits the toxicity of *w*Bm0076 expression, we considered the possibility that *w*Bm0076 would localize to cortical actin patches to regulate their formation *in vivo*. Accordingly, we constructed a *w*Bm0076-mRuby expression construct that still maintained toxicity (and thus, activity) upon expression (Fig 6B). In a yeast strain harboring Abp140-3xGFP, which also localizes to cortical actin patches (Fig 4B), expression of *w*Bm0076-mRuby induces the formation of cortical actin patches and concomitant loss of actin cables previously observed (Fig 6C, compare to Fig 4B). Additionally, we observe colocalization of *w*Bm0076 with these Abp140-3xGFP-positive punctae, suggesting that *w*Bm0076 localizes to cortical actin patches to regulate actin dynamics and may do so through direct interactions with yeast proteins directly involved in actin patch formation.

Based upon the finding that *abp1Δ* strains limit the toxicity of *w*Bm0076 expression, *w*Bm0076-mRuby localizes to cortical actin patches, and the knowledge that Abp1p is intimately involved in activating Arp2/3 during endocytosis, we find it likely that the toxicity of *w*Bm0076 in yeast is directly related to its ability to alter actin dynamics *in vivo*. Given the conservation of the regulation of actin dynamics across eukaryotes, these data suggest that *w*Bm0076 may be important for the reorganization of actin structures in the nematode.

## Discussion

Though some progress has been made in understanding the *Wolbachia*:host relationship at the molecular level, much remains unknown about the mechanisms by which *Wolbachia* persists in its nematode or insect hosts. The use of *S*. *cerevisiae* as a highly tractable surrogate eukaryotic host provides an avenue to rapidly screen *w*Bm putative T4SS effectors that may manipulate eukaryotic cell physiology and membrane trafficking pathways likely required for the persistence of *Wolbachia* in host cells. Historically, the *S*. *cerevisiae* model has successfully identified virulence factors from human pathogens that, upon expression in yeast, induce a growth defect and alter eukaryotic physiology, as is the case with the identification of secreted proteins from a variety of pathogenic bacteria, including *Chlamydia trachomatis*, *Legionella pneumophila*, *Pseudomonas aeruginosa*, and *Salmonella enterica* [27, 29, 77, 78]. Recently, *S*. *cerevisiae* was used to identify fourteen candidate effectors of the *Wolbachia* endosymbiont of *Drosophila melanogaster*, *w*Mel, with each candidate effector inducing growth defects in *S*. *cerevisiae* [79]. Collectively, these data have verified that *S*. *cerevisiae* is a valuable tool to evaluate the molecular function of a number of *Wolbachia*-secreted proteins. Therefore, we have employed *S*. *cerevisiae* to screen 47 predicted *w*Bm effectors to identify those that manipulate eukaryotic physiology. Due to the ability of some of these proteins to induce growth defects and alter the cellular processes in *S*. *cerevisiae* (Table 2), we propose that they may function as secreted effectors of *w*Bm and may be necessary for germline cell invasion and persistence of *w*Bm in its nematode host. Strikingly, two of these proteins–*w*Bm0076 and *w*Bm0152—are particularly strong modulators of yeast physiology.

**Table 2:** Summary of aberrant yeast phenotypes observed in this study.

Growth^a^	Growth with Zn/caffeine^a^^,^^b^	Vacuole morphology^c^	Endosome:vacuole cargo trafficking
*w*Bm0014 (strong)	*w*Bm0057 (strong)	*w*Bm0076	*w*Bm0152
*w*Bm0032 (strong)	*w*Bm0064 (strong)	*w*Bm0114	
*w*Bm0044 (strong)	*w*Bm0213 (strong)	*w*Bm0152	
*w*Bm0070 (weak)	*w*Bm0277 (strong)	*w*Bm0447	
*w*Bm0076 (strong)	*w*Bm0287 (strong)		
*w*Bm0114 (weak)	*w*Bm0447 (strong)		
*w*Bm0222 (weak)			
*w*Bm0284 (strong)			

^a^Inhibition of cell growth on indicated medium denoted as strong (cell titer three orders of magnitude lower in a serial dilution) or weak (two orders of magnitude), when compared to no induction controls.

^b^Inhibition of cell growth on medium containing ZnCl_2_ and caffeine (Fig 1B), but not on medium lacking these additions.

^c^Proteins capable of producing aberrant vacuolar morphologies in ≥30% of the cells examined.

*w*Bm0152, conserved across *Wolbachia* isolates of diverse hosts, is a predicted peptidoglycan associated lipoprotein (PAL) that was previously identified as a component of the *wBm* secretome [80]. PAL proteins are thought to be intimately involved in regulating Gram-negative cell envelope integrity via interactions with both periplasmic peptidoglycan and the outer membrane [81]. Relatively recent work has found, however, that PAL proteins can exist in a so-called ‘dual conformation,’ where a population of these proteins are surface-exposed [82]. This particular conformation creates the possibility that these proteins may also be involved in extracellular contacts. Indeed, previous work by Melnikow and coworkers have shown that *w*Bm0152 was found to bind actin and was hypothesized to be an important link between *Wolbachia* cells and host actin/tubulin during endosymbiosis, even though direct binding between *w*Bm0152 and actin in solution was not directly assayed [17]. In our study, we find that expression of *w*Bm0152 in *S*. *cerevisiae* induces a striking endosomal trafficking defect, as seen by aberrant “class E” vacuole morphologies, and in the mislocalization of the endosomal cargo proteins (CPS and Sna3p) to an enlarged prevacuolar compartment. This phenotype is reminiscent of yeast strains defective in endosomal sorting complex required for transport (ESCRT) complex proteins, which are involved in vacuolar protein sorting, Golgi protein recycling, and membrane repair/remodeling pathways [56]. Because the membrane comprising *Wolbachia* replicative vesicles in *Drosophila* appear to originate from the host Golgi [38], and because *w*Bm0152 seems to delay or block the delivery of Golgi-derived cargo to the vacuole in yeast, it is tempting to speculate that *w*Bm0152 may play a role in either the biogenesis of the *w*Bm membrane-bound compartment, or preventing the degradation of the *Wolbachia*-containing vacuole by interfering with its fusion with the nematode lysosome.

*w*Bm0076 is a WAS family protein that likely promotes actin cytoskeletal rearrangements and has sequence homology to RickA, a protein expressed by *Rickettsia conorii* that is known to activate Arp2/3 and drive the polymerization of host actin to support bacterial intracellular motility during infection, similar to that of *Listeria monocytogenes* [67, 83]. In eukaryotes, Arp2/3 is a protein complex that is associated with cortical actin patches and aids in actin arrangement for the nucleation of branched actin filaments that drive processes like endocytosis and cellular motility [65, 66]. In yeast, it has been shown that the conserved Abp1p protein interacts with both actin and Arp2/3 complex to initialize actin polymerization in *S*. *cerevisiae* [70, 84], specifically localizing to cortical actin patches and initiating endocytic vesicle formation and actin patch disassembly through its recruitment of other kinases [85, 86]. Our data shows *w*Bm0076 expression induces yeast cell lysis, in a manner similar to strains lacking F-actin [87]. Therefore, *w*Bm0076 may induce yeast cell lysis through the elimination of F-actin structures by either enhanced turnover of F-actin, or through the enhanced polymerization of branched actin structures and the concomitant decrease in monomeric actin required to maintain F-actin. Due to the fact that we have also identified a genetic interaction between Abp1p and *w*Bm0076, it is possible limiting excess Arp2/3 activation (and hence, excessive branched actin formation) by both Abp1p and *w*Bm0076 independently, is sufficient to limit cell lysis upon *w*Bm0076 expression. Alternatively, Abp1p may be the direct target of *w*Bm0076 *in vivo* to regulate actin polymerization since the ability of Abp1 and N-WASP to directly interact to stimulate Arp2/3 activity has previously been shown in mammalian neuronal cells [88]. Interestingly, it has been shown that *Wolbachia* induces a reorganization of cellular F-actin structures during invasion of the germline in female nematodes, thus ensuring the vertical transmission of *Wolbachia* to offspring [89]. It is therefore possible that *w*Bm0076 plays a vital role in this particular process, as actin “comet tails” have not, as of yet, been detected to play a role in the mobility of *Wolbachia* in *B*. *malayi* [90]; *B*. *malayi* contains the Abp1p homolog Bm4914 (20% end-to-end identity; 32% similar to yeast Abp1p), which could serve as the target of *w*Bm0076 in the nematode. Interestingly, profilin mutants of *Drosophila* are incapable of efficient maternal passage of *Wolbachia* to offspring [90], and the *Wolbachia* parasite of *Drosophila* (*w*Mel) produces at least one other putative secreted effector, WD0830, capable of bundling actin filaments *in vivo* and *in vitro* via its α-synuclein domain [91]. It is also important to note that the *w*Bm0076 homolog in *w*Mel, WD0811, while containing a clear WH2 domain, lacks the conserved C and A domains usually found in WASP-family proteins (Fig 4); WD0811 therefore may manipulate actin polymerization in *Drosophila* in a manner distinct from that of *w*Bm0076. Additionally, WD0811 has been previously been shown to be translocated through a surrogate T4SS [92], thus providing additional evidence to support the hypothesis that *w*Bm0076 in an authentic T4SS effector of *wBm*. The data presented in this study provide additional mounting evidence that *Wolbachia* modulates host actin for successful transmission to offspring.

Recently, researchers have carried out a similar screen of eighty-four putative *w*Mel effectors in yeast [79]. Of those eighty-four, twelve (based on sequence homology) were also screened in our study and three (*w*Bm0076/WD0811; 36% identical, *w*Bm0287/WD0566; 40% identical, *w*Bm0447/WD0438; 44% identical) were moderately to severely toxic to yeast cells as determined by induced growth defects in the presence or absence of stressors. Notably, both WD0811 and WD0438 were identified by Rice et al. as candidate effectors of *w*Mel due to induced growth defects in yeast [79]. *w*Mel proteins are more similar to *Wolbachia* endosymbionts of arthropods within its same or similar clade than it is to *w*Bm [79]; *w*Bm and *w*Mel have 696 orthologous proteins and similar metabolic capacities that likely differ due to host and the relationship *Wolbachia* maintains with such host [14]. The use of yeast to identify putative effectors from *Wolbachia* endosymbionts of distinct hosts, and specifically homologous putative effectors, confirms the validity of such a screen for use as an initial characterization of protein functions in a genetically intractable system. In this work, we have furthered the characterization of two putative effectors of *w*Bm that appear to have drastic effects on yeast cell biology, thus providing additional insight into their physiological function to support the intracellular survival of *Wolbachia* in *B*. *malayi*. Although we find individual *w*Bm proteins can manipulate yeast growth and trafficking pathways, it is most likely a concerted effort of many secreted proteins is required to maintain both the intracellular colonization of *w*Bm and survival of *B*. *malayi*. Our studies demonstrate the successful use of *S*. *cerevisiae* as a host system to identify putative effectors that may shed light on their roles in manipulating the cellular physiology of *B*. *malayi* to help maintain this essential relationship.

## Materials and methods

### Yeast strains and plasmid constructions

For all growth analyses and microscopic visualization studies, yeast strain BY4742 (MATα *his3Δ*1 *leu2Δ*0 *lys2Δ0 ura3Δ0*) was used for all studies. In order to create yeast strains that activate *GAL1* promoters via the addition of β-estradiol, strains were transformed with linearized pAGL (a gift from Dr. Daniel Gottschling, University of Washington), which introduces the gene encoding for the Gal4-estrogen receptor-VP16 (GEV) chimeric protein into the *leu2Δ*0 locus [93]. S288C yeast strains expressing either Abp1p-RFP or Abp140-3xGFP were a kind gift from Dr. Bruce Goode (Brandeis University).

Hypothetical or eukaryotic-domain containing *Wolbachia* proteins such as ankyrin repeats or Ser/Thr kinase motifs were searched for using BLAST against the NCBI database. Proteins highly related to eukaryotes rather than bacteria were selected for this study. Coiled coil domain containing proteins were predicted using an online tool at: https://npsa-prabi.ibcp.fr/cgi-bin/npsa_automat.pl?page=npsa_lupas.html [94]. *Rickettsia sibirica* proteins that interact with different components of its T4SS identified in a bacterial two-hybrid system [36] and proteins that potentially co-transcribed with components of T4SS were used to as query to search using TBLASTN against the *w*Bm genome to identify the potential homolog in filarial *Wolbachia*. *Wolbachia* proteins previously identified in excretory-secretory (ES) products from *B*. *malayi* adult worms and microfilaria [37] were also selected. Domain structures of *w*Bm0076 and orthologs were determined with HHpred (MPI bioinformatics Toolkit, https://toolkit.tuebingen.mpg.de/#/tools/hhpred).

*Wolbachia* genes encoding for putative T4SS-secreted proteins were cloned into the galactose-inducible yeast expression vector, pYES2/NT A via the USER (uracil-specific excision reagent) cloning system developed at NEB. Briefly, gene-specific PCR primers were designed to clone the predicted *w*Bm open reading frame into the vector expressing an amino-terminal Xpress epitope tag; these primers (S1 Table) contained a linker complementary to pYES2/NT A, with an additional uracil, and were used to amplify the target gene with *PfuTurbo* C_x_ hotstart DNA polymerase (Stratagene). PCR products were then treated with the USER Enzyme (New England Biolabs) to create unique 3´ single-stranded extensions which anneal and ligate to linearized pYES2/NT A, which had been previously amplified with vector-specific primers containing a complementary linker to the insert gene of interest.

In order to utilize the *URA3*^*+*^ pGO45 and pMM134 plasmids, which express GFP-CPS or Sna3p-GFP, respectively [57, 95], for yeast endosome:vacuole transport studies in the presence of another *URA3*^*+*^ yeast expression plasmid harboring the putative *w*Bm effector, pGO45 and pMM134 were first converted from *URA3*^*+*^ to *LYS2*^*+*^ via homologous recombination methods by digesting pM2660 (ATCC) with HindIII [96], and transforming yeast strains harboring either pGO45 or pMM134 with this digest. Growth was selected on CSM medium + 2% glucose lacking lysine. Colonies from these plates were screened for lack of growth on CSM + 2% glucose medium without uracil. Plasmids from phenotypically correct strains were isolated, reintroduced into the appropriate yeast background, and confirmed for function via fluorescence microscopy.

To create the wBm0076-mRuby expressing pYES2NTA plasmid, the yomRuby2 gene was amplified from the plasmid pFA6a-link-yomRuby2-SpHis5 [97] using primers 5’- AGCTTTTCTTATAAAACAATTGATGGTGTCCAAAGGAGAGGAG and 5’- AGGGATAGGCTTAGCTGCAATTTACTTATACAATTCATCCATA, containing homology to both the C-terminus of the wBm0076 gene and the pYES2NTA-*w*Bm0076 vector. BY4742 was co-transformed with pYES2NTA-wBm0076, previously digested with PmeI, and the mRuby2 amplicon and were plated to CSM-uracil to select for gap-repaired plasmids. Transformants were screened for red fluorescence via microscopy, and the resultant plasmid was purified and sequenced for confirmation (Eton Bioscience Inc).

### Cell lysis and western blotting

β-estradiol responsive yeast strains harboring *GAL*-inducible pYES2/NT A control plasmid or pYES2/NT A containing an individual *w*Bm open reading frame were grown to saturation in CSM-uracil medium, subcultured to CSM-uracil supplemented with 1 μM β-estradiol and grown for 6h at 30°C. 5.0 OD_600_ units were harvested and protein was extracted in lysis buffer (0.1 M NaOH, 2% SDS, 2% β-mercaptoethanol, 0.05 M EDTA-NaOH pH 8.0) and boiled 5 minutes prior to the addition of 0.1M acetic acid. Equal volumes of extracts were separated on 13% SDS-PAGE gels and immunoblotted for *w*Bm proteins using the commercially-available anti-Xpress monoclonal antibody (1:1000, Thermo Fisher Scientific) for 1 h, using blocking buffer containing 0.5% Tween and 5% dry non-fat milk. Goat anti-mouse IgG secondary antibody conjugated to HRP (1:20000, Thermo Fisher Scientific) was applied to the membrane for 1 h, washed, and developed with SuperSignal West Pico PLUS chemiluminescent substrate (Thermo Scientific).

### Microscopy

For evaluation of vacuole morphologies, β-estradiol responsive yeast strains harboring either control pYES2/NT A vector or pYES2/NT A WSPs were grown to saturation in selective medium at 30°C, subcultured to fresh media with or without 1 μM β-estradiol and grown for an additional 6 hours. After 6 hours, the entire culture was harvested via centrifugation, suspended in 50 μL CSM lacking uracil, and FM4-64 was added to 3 μM. Cells were incubated at 30°C for 20 minutes, harvested, washed, then suspended in 5 mL fresh medium. This culture was incubated for an additional 90 minutes at 30°C, harvested, washed, and suspended in 25 μL CSM medium lacking uracil. Cell suspensions were mounted to slides that had been pre-treated with a 1:1 mixture of polylysine (10% w/v):concanavalin A (2 mg/ml) solution. Cells were visualized using a Nikon Ti-U fluorescence microscope, and images were processed using the Fiji software package [98, 99].

### Statistical analysis

Statistical analysis was performed within the Prism software package (GraphPad Software, v. 6.0b). Column statistics were performed via a 1-way ANOVA Repeated Measures test and Holm-Bonferroni post-test. Where noted in figures, ns = P > 0.05 (not significant); (*) = P ≤ 0.05; (**) = P ≤ 0.01; (****) = P ≤ 0.0001.

## Supporting information

S1 Table Primers used in this study. (DOCX)

S1 Fig Analysis of *wBm* effector protein expression in yeast. BY4742 yeast strains genetically modified with GEV for β-estradiol-dependent induction of *GAL* promoters (Materials and Methods) were assayed for individual putative *w*Bm effector expression via anti-Xpress immunoblot (Materials and Methods). Images shown are representative of three independent replicates.
(PDF)

S2 Fig Most individual *wBm* effector genes do not inhibit yeast growth upon expression. BY4742 yeast strains genetically modified with GEV for β-estradiol-dependent induction of *GAL* promoters (Materials and Methods) and harboring the *GAL-*inducible control plasmid pYES2/NT A, or pYES2/NT A containing the specified *wB*m open reading frame were grown to saturation in CSM-uracil medium containing 2% glucose, and each culture was diluted to OD_600_ = 1.0 in sterile 0.9% NaCl. 10-fold serial dilutions were spotted onto CSM-uracil containing 2% glucose with and without 1 μM β-estradiol or 7.5 mM ZnCl_2_/5 mM caffeine. Plates were incubated for 48 or 72 h at 30°C; results are representative of three independent experiments.
(PDF)

S3 Fig Effects of *wBm* effector gene expression on yeast vacuole morphology. BY4742 yeast strains modified with GEV for β-estradiol-dependent induction of *GAL* promoters (Materials and Methods), and harboring *GAL*-inducible pYES2/NT A control plasmid or pYES2/NT A containing an individual *w*Bm open reading frame were grown to saturation in CSM-uracil medium, subcultured to CSM-uracil supplemented with 1 μM β-estradiol, and grown for 6h at 30°C. Cells were stained for 20 minutes with 10 μM FM4-64 at 30°C, followed by a 1.5 h chase in CSM-uracil at 30°C. Cells were visualized and representative crops from three independent experiments were generated; bar = 5 μ.
(PDF)

S4 Fig CPS-GFP is generally not mislocalized in yeast strains expressing *wBm* genes. BY4742 yeast strains modified with GEV for β-estradiol-dependent induction of *GAL* promoters (Materials and Methods), and harboring pGO45 GFP-CPS plasmid in addition to pYES2/NT A control plasmid or pYES2/NT A harboring an individual *w*Bm open reading frame were grown to saturation in CSM-lysine-uracil medium. Cells were subcultured to CSM-lysine-uracil with 1 μM β-estradiol and grown for 6 h at 30°C. Cells were stained with 10 μM FM46-4 for 20 minutes at 30°C, chased for 1.5 h in CSM-lysine-uracil medium at 30°C, then visualized. Representative crops from two independent experiments are shown; bar = 3 μ.
(PDF)

S5 Fig GFP-Sna3 is generally not mislocalized in yeast strains expressing *wBm* genes. BY4742 yeast strains modified with GEV for β-estradiol-dependent induction of *GAL* promoters (Materials and Methods) and harboring the Sna3-GFP plasmid in addition to pYES2/NT A control plasmid, or pYES2/NT A harboring an individual *w*Bm open reading frame, were grown to saturation in CSM-lysine-uracil medium. Cells were subcultured to CSM-lysine-uracil with 1 μM β-estradiol and grown for 6 h at 30°C. Cells were stained with 10 μM FM46-4 for 20 minutes at 30°C, chased for 1.5 h in CSM-lysine-uracil medium at 30°C, then visualized. Representative images from two independent experiments are shown; bar = 3 μ.
(PDF)

S6 Fig Deletions of genes involved in yeast actin dynamics do not reduce the toxicity of *w*Bm0076 expression. BY4742 yeast strains deleted for the indicated gene and harboring either pYES2/NT A or pYES2/NT A *w*Bm0076 (0076) were grown overnight in CSM medium lacking uracil. Cultures were diluted to an OD_600_ = 1.0 in sterile 0.9% NaCl, then spotted in 10-fold dilutions on plates containing 1% raffinose and either 2% glucose or 2% galactose to induce *WBM0076* expression. Plates were incubated for 72 h at 30°C and imaged; results are representative of three independent experiments.
(PDF)
