# Supplementary material for: Identification of putative effectors of the Type IV secretion system from the Wolbachia endosymbiont of Brugia malayi
Source: PLoS One. 2018 Sep 27;13(9):e0204736. doi: 10.1371/journal.pone.0204736 (PMC6160203; doi:10.1371/journal.pone.0204736)
Supplement: S1 Table — (DOCX) [file pone.0204736.s001.docx]

Supplemental Information for

**Identification of putative effectors of the Type IV secretion system from the *Wolbachia* endosymbiont of *Brugia malayi***

Emily M. Carpinone^¶1^, Zhiru Li^¶2^, Michael K. Mills^1^, Clemence Foltz^2^, Emma R. Brannon^1^, Clotilde K.S. Carlow^2^, and Vincent J. Starai^1,3*^

^1^Department of Microbiology, University of Georgia, Athens, GA, USA

^2^Division of Genome Biology, New England Biolabs, Ipswich, MA, USA

^3^Department of Infectious Diseases, University of Georgia, Athens, GA, USA

*Corresponding author

E-mail: [vjstarai@uga.edu](mailto:vjstarai@uga.edu) (VJS)

^¶^These authors contributed equally to this work.

**S1 Table. Primers used in this study.**

| Gene | Forward | Reverse |
| --- | --- | --- |
| pYES2NT | ATTGCAGC/ideoxyU/AAGCCTATCCCTAACCCTCTCCTCGGTCT | AGCCTCCT/ideoxyU/TACCTTATCGTCATCGTCGTACAGATCCCG |
| *WBM0014* | AAGGAGGC/ideoxyU/ATGCTCGAATGTGAAAATTTATCCTGTATT | AGCTGCAA/ideoxyU/TCAGCTATAACTATTGAAATTACACACGTC |
| *WBM0032* | AAGGAGGC/ideoxyU/ATGATGAACACGGATGAAACTATTTTTGC | AGCTGCAA/ideoxyU/TCACTTGCCTACACAGAAGTTATTGAATAT |
| *WBM0044* | AAGGAGGC/ideoxyU/ATGTCAAATAAAATAGGTTTTTTGGCTGTT | AGCTGCAA/ideoxyU/TTATTGTGCGGAAGCTCTTCGGTAC |
| *WBM0057* | AAGGAGGC/ideoxyU/ATGCAAGTAGTAGAAATAGTTATACGTAATAG | AGCTGCAA/ideoxyU/CTAATCCTTTATGCACTCAATGATCTTATT |
| *WBM0064* | AAGGAGGC/ideoxyU/ATGAGTTACGACGTAATCATTTCAGGT | AGCTGCAA/ideoxyU/TTACACAAATCCCATAGCGTGCCGGAT |
| *WBM0070* | AAGGAGGC/ideoxyU/ATGGATTGGTTATTGGTTTCAATATTATCGATA | AGCTGCAA/ideoxyU/TTAATCAATTGCTGTTACAGTATTATCAGTTTTTA |
| *WBM0076* | AAGGAGGC/ideoxyU/ATGCGTATATCTAATTTTGGACCTTTAATTGC | AGCTGCAA/ideoxyU/CTATTGTTTTATAAGAAAAGCTCTAGATTCTTT |
| *WBM0100* | AAGGAGGC/ideoxyU/ATGATGAAAAGATCTATTGTTTTTGATGCAT | AGCTGCAA/ideoxyU/CTAAAAATTAAATATTAAGCCAATTTCAATGTTATGG |
| *WBM0114* | AAGGAGGC/ideoxyU/ATGCCTAAATTATCAATTGTAGTTGCTCC | AGCTGCAA/ideoxyU/TCATCTCTCATAGTGTCTCTTAAACTTTTC |
| *WBM0152* | AAGGAGGC/ideoxyU/ATGTGGAGTAGATTGGTTGCAATGTGC | AGCTGCAA/ideoxyU/CTATTTTTTCATTCCAGAAAATGAAAACTC |
| *WBM0164* | AAGGAGGC/ideoxyU/ATGTCTAGCCAAAAGGGTGTAGATCTG | AGCTGCAA/ideoxyU/TCATGAAGTGCCTCTGTATGTGCTG |
| *WBM0165* | AAGGAGGC/ideoxyU/ATGGAAGAAGCATGCAAAACGTGTG | AGCTGCAA/ideoxyU/TCAATCTTTTTTGCACATTTTTTTATCTT |
| *WBM0181* | AAGGAGGC/ideoxyU/ATGGACTTAAATAAATTTACCGAAAAAGCA | AGCTGCAA/ideoxyU/TTAAACCTTTTTAACTAAGATTTCATTATTAAAAGC |
| *WBM0193* | AAGGAGGC/ideoxyU/ATGAACATTGAAAATATACAACAAGAATTTTTTCC | AGCTGCAA/ideoxyU/TTATATTGATATACCTTTACCTTTTTCTTGTTGTTG |
| *WBM0209* | AAGGAGGC/ideoxyU/GTGGATACTGTTTATGTGAAATATGTTTGTCAAG | AGCTGCAA/ideoxyU/TTATCCAACACACTTAGATTTCTGAGCC |
| *WBM0213* | AAGGAGGC/ideoxyU/ATGAAAATAAAGCTGTTTTTTATTTTCACTCTACTGT | AGCTGCAA/ideoxyU/TTATTTAATAGATAAGCTATCTAAGTCAAATTTTTGCTT |
| *WBM0222* | AAGGAGGC/ideoxyU/ATGTTCATTTCTGAAGTTTTTGCGGCA | AGCTGCAA/ideoxyU/TTATGAAGTATTTTTACCTTTACTATCTTTCCCCT |
| *WBM0277* | AAGGAGGC/ideoxyU/ATGAATATTAATATCTTTGTTTTTTTAGCTTTATCTTTT | AGCTGCAA/ideoxyU/TCAATTTATCTCAAGCTTAAAGTCTTCCG |
| *WBM0284* | AAGGAGGC/ideoxyU/ATGATGAGCAATAAAAAAACATTAGCGGTTA | AGCTGCAA/ideoxyU/TTAAACCTTGCTAGCAAAATGAAAAGTAAG |
| *WBM0287* | AAGGAGGC/ideoxyU/ATGCATGTTCACAAGAATGTTGTAGAG | AGCTGCAA/ideoxyU/TTATTCCTTGATTAAGAAAAATTCTACAACGTT |
| *WBM0290* | AAGGAGGC/ideoxyU/ATGAGTATATTAGACAAATTGGTAATCCTGC | AGCTGCAA/ideoxyU/TTAAAACAATATTCTAAAAAACTTTTCTACGTAATT |
| *WBM0307* | AAGGAGGC/ideoxyU/ATGAGTGGCATACCAAAAGGTATAAGAC | AGCTGCAA/ideoxyU/CTATTTTATCACTGGTGGTTTTTCGAAAG |
| *WBM0384* | AAGGAGGC/ideoxyU/ATGTCCAATTATATACAAAATAGCGGAACTA | AGCTGCAA/ideoxyU/TTAGATTAAGCTATGATCATGGTAAATATGA |
| *WBM0394* | AAGGAGGC/ideoxyU/aTGTTAGATCATAGTACTAGCTATGGGC | AGCTGCAA/ideoxyU/TTAGTTATTGACGACAGCAAAATATAAAGGA |
| *WBM0430* | AAGGAGGC/ideoxyU/ATGCGTAGTTTTTTTGTATTTCTAATATTTTTTTCA | AGCTGCAA/ideoxyU/TCACTTTACGTAGCATAAATCATGCATTT |
| *WBM0432* | AAGGAGGC/ideoxyU/ATGCATTATAAAAAGTTTTTTTCAGCAACCG | AGCTGCAA/ideoxyU/TTAGAAATTAAACGCTATTCCAGCTTCT |
| *WBM0447* | AAGGAGGC/ideoxyU/ATGAGTATAGATATAACTACACTAACTACTAATACAG | AGCTGCAA/ideoxyU/TCATCGCGCCAGAGAAGAGGGTTTATT |
| *WBM0452* | AAGGAGGC/ideoxyU/ATGAATGCTGCTCTATACCATAAAGAAT | AGCTGCAA/ideoxyU/CTATGAATGATGAATCAGTATTGCTTTAAAAA |
| *WBM0482* | AAGGAGGC/ideoxyU/ATGAATAGTACTTGGCAAAAATGCATAGG | AGCTGCAA/ideoxyU/TCATGAGACATATTGTAATAAGTGATGATATATTAT |
| *WBM0484* | AAGGAGGC/ideoxyU/ATGTTAGAGGCGTTAAAGAGAGTAGGAAC | AGCTGCAA/ideoxyU/TCAATTATGGATTTGTGAAACACATTTATCTTGGTCTC |
| *WBM0491* | AAGGAGGC/ideoxyU/ATGGTCCCCTCTGTAAGGAGAAGCA | AGCTGCAA/ideoxyU/CTACTGTTTAAGCTTAAAAAATGTATATGATAGTG |
| *WBM0506* | AAGGAGGC/ideoxyU/ATGCGTAAGGTTTTCATCTATACAATAATTTCTT | AGCTGCAA/ideoxyU/CTAATTAATATTTTCATTCTTGGTTAGTTTGGTG |
| *WBM0582* | AAGGAGGC/ideoxyU/ATGAGAAGTGTTTTATACTTTACGTTGTTATTTG | AGCTGCAA/ideoxyU/TCAGTCTTCATTGCATTGTAATTCATTATCT |
| *WBM0665* | AAGGAGGC/ideoxyU/TTGCACATCGTTACTTTGCATGGTAA | AGCTGCAA/ideoxyU/TCATACTATTTGTAGTAAAAATCTGTAACATTTGC |
| *WBM0666* | AAGGAGGC/ideoxyU/ATGGTGACCTTGAGTGTAAGAGAAGCTTTATGTA | AGCTGCAA/ideoxyU/TTATTTTTTTCTAAAACAGACCTGATGCACAGT |
| *WBM0671* | AAGGAGGC/ideoxyU/ATGTATAAGGCTTTAATCACGTGCTTTA | AGCTGCAA/ideoxyU/TTACTGTTCTACAGTATTCTCTTTGAGGT |
| *WBM0672* | AAGGAGGC/ideoxyU/ATGACTAGTAACTTTGGAACTAAATTTTCTGC | AGCTGCAA/ideoxyU/TCACATGTTTGTGCAGATATGAATCG |
| *WBM0709* | AAGGAGGC/ideoxyU/ATGAAAGAACAAAAAATACAAGCTTTCGAA | AGCTGCAA/ideoxyU/CTACATCCACTTAACAAGAGGTGGCAT |
| *WBM0711* | AAGGAGGC/ideoxyU/ATGCTTTTCAATTCTATCGCTTTTGG | AGCTGCAA/ideoxyU/CTAATCCACCCTCAATTCCTCGGC |
| *WBM0736* | AAGGAGGC/ideoxyU/ATGACTAGAAAAATTATACTACAATTGCTTATT | AGCTGCAA/ideoxyU/TTACTCAACAGTAGAGTGATGTGCTGCTATTT |
| *WBM0748* | AAGGAGGC/ideoxyU/TTGGCTTACGCCATACGTAAGCAG | AGCTGCAA/ideoxyU/TTAAAAAAGGATCTTGACTGGCAGTAGTTCC |
| *WBM0749* | AAGGAGGC/ideoxyU/ATGGAAAGTGTAAAACTTAATTTTTTATTGATA | AGCTGCAA/ideoxyU/CTATAGCCCTAATAATTTCTTTTTGATTTCTTCCT |
| *WBM0751* | AAGGAGGC/ideoxyU/ATGCACATTCAATTGAATAAAGTTTTGATTTT | AGCTGCAA/ideoxyU/CTACAAGTGATACTTAATTTTTATCTTTTTTTTACTAT |
| *WBM0752* | AAGGAGGC/ideoxyU/GTGGATGATAATGTAGCGATAGTTTTTAATTTAACA | AGCTGCAA/ideoxyU/TTAAACATGCAGTAGGCTTCTTTCATTATTAATAA |
| *WBM0772* | AAGGAGGC/ideoxyU/ATGGCAGAAGAGATTAACATTATGAACG | AGCTGCAA/ideoxyU/CTATCTTGATTGTGCTTCTCCTTGTGC |
| *WBM0791* | AAGGAGGC/ideoxyU/ATGCTGCGTATTATTGCAGGAAAGTATC | AGCTGCAA/ideoxyU/TTAAGTTGATAGAGAAAGAAAAATTATTCTTGCTA |
| *WBM0792* | AAGGAGGC/ideoxyU/GTGAATAGGATAACAATGAAAGCTAGTAAAAA | AGCTGCAA/ideoxyU/TCAATCACTTTTTTTTGATTTATCAACTATATCGA |
