# Supplementary figures and images for: Identification of putative effectors of the Type IV secretion system from the Wolbachia endosymbiont of Brugia malayi
Source: PLoS One. 2018 Sep 27;13(9):e0204736. doi: 10.1371/journal.pone.0204736 (PMC6160203; doi:10.1371/journal.pone.0204736)

Figure S1

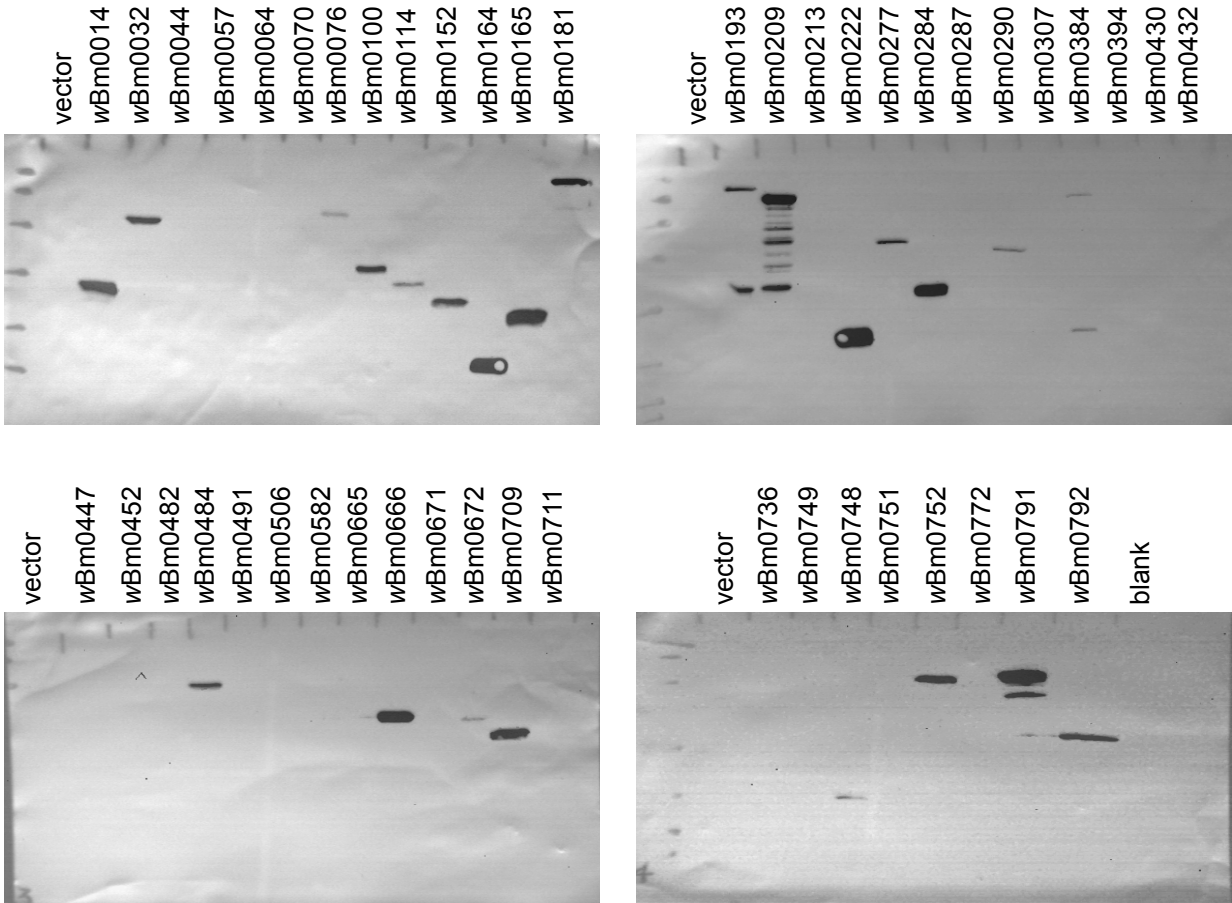

Supplement: S1 Fig — BY4742 yeast strains genetically modified with GEV for β-estradiol-dependent induction of GAL promoters (Materials and Methods) were assayed for individual putative wBm effector expression via anti-Xpress immunoblot (Materials and Methods). Images shown are representative of three independent replicates. (PDF) [file pone.0204736.s002.pdf]

Figure S2

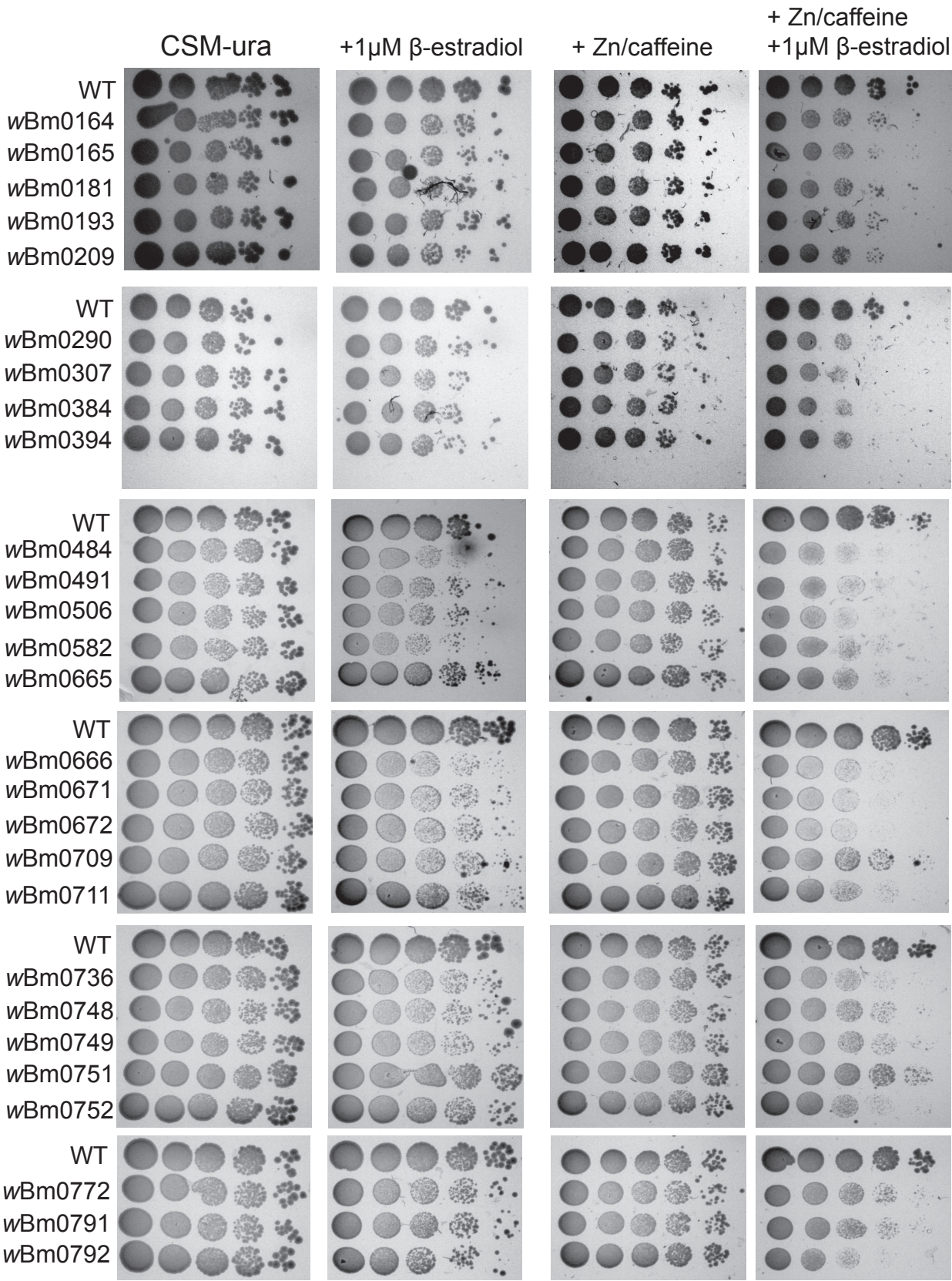

Supplement: S2 Fig — BY4742 yeast strains genetically modified with GEV for β-estradiol-dependent induction of GAL promoters (Materials and Methods) and harboring the GAL-inducible control plasmid pYES2/NT A, or pYES2/NT A containing the specified wBm open reading frame were grown to saturation in CSM-uracil medium containing 2% glucose, and each culture was diluted to OD600 = 1.0 in sterile 0.9% NaCl. 10-fold serial dilutions were spotted onto CSM-uracil containing 2% glucose with and without 1 μM β-estradiol or 7.5 mM ZnCl2/5 mM caffeine. Plates were incubated for 48 or 72 h at 30°C; results are representative of three independent experiments. (PDF) [file pone.0204736.s003.pdf]

# Figure S3

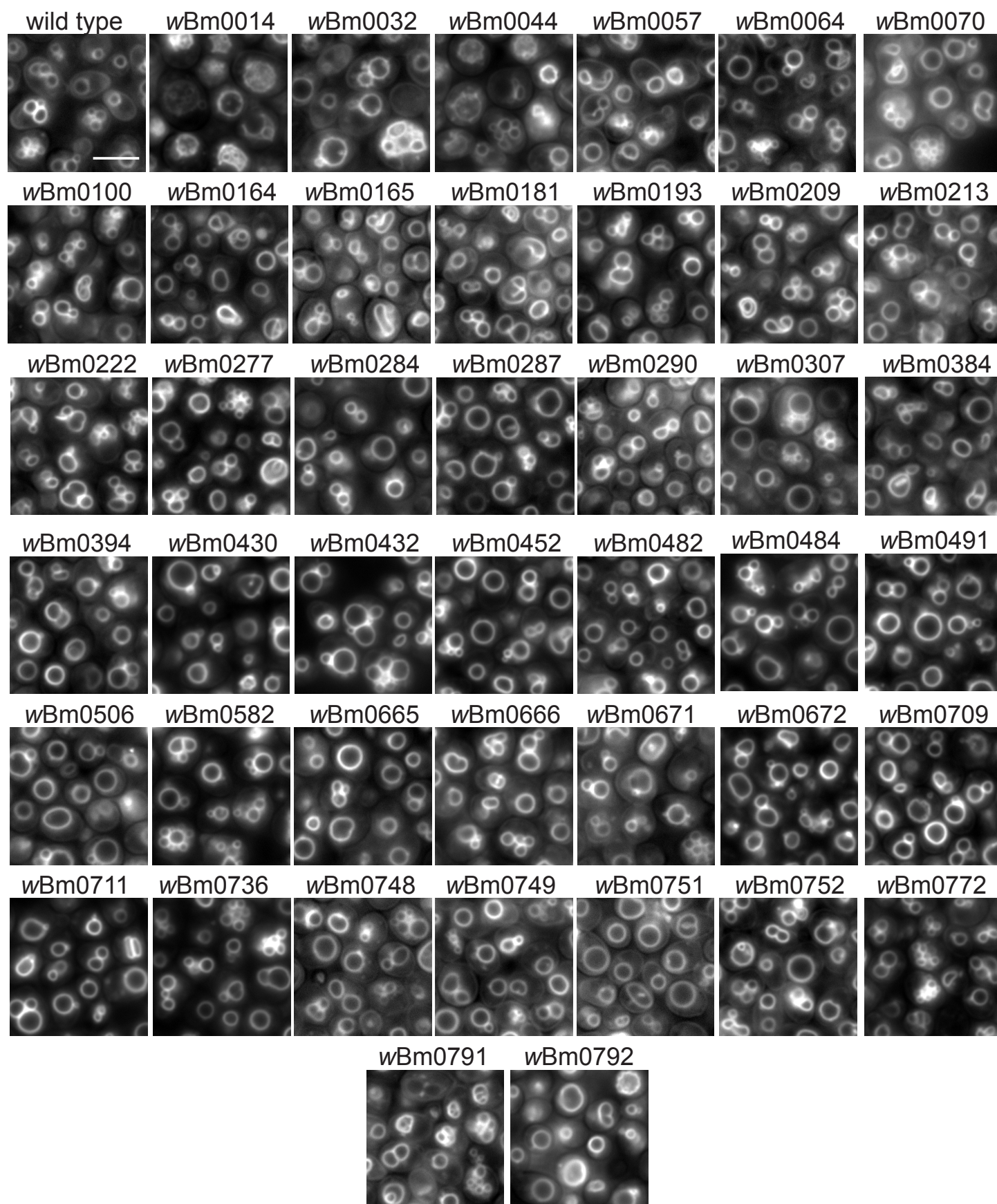

Supplement: S3 Fig — BY4742 yeast strains modified with GEV for β-estradiol-dependent induction of GAL promoters (Materials and Methods), and harboring GAL-inducible pYES2/NT A control plasmid or pYES2/NT A containing an individual wBm open reading frame were grown to saturation in CSM-uracil medium, subcultured to CSM-uracil supplemented with 1 μM β-estradiol, and grown for 6h at 30°C. Cells were stained for 20 minutes with 10 μM FM4-64 at 30°C, followed by a 1.5 h chase in CSM-uracil at 30°C. Cells were visualized and representative crops from three independent experiments were generated; bar = 5 μ. (PDF) [file pone.0204736.s004.pdf]

# Figure S4

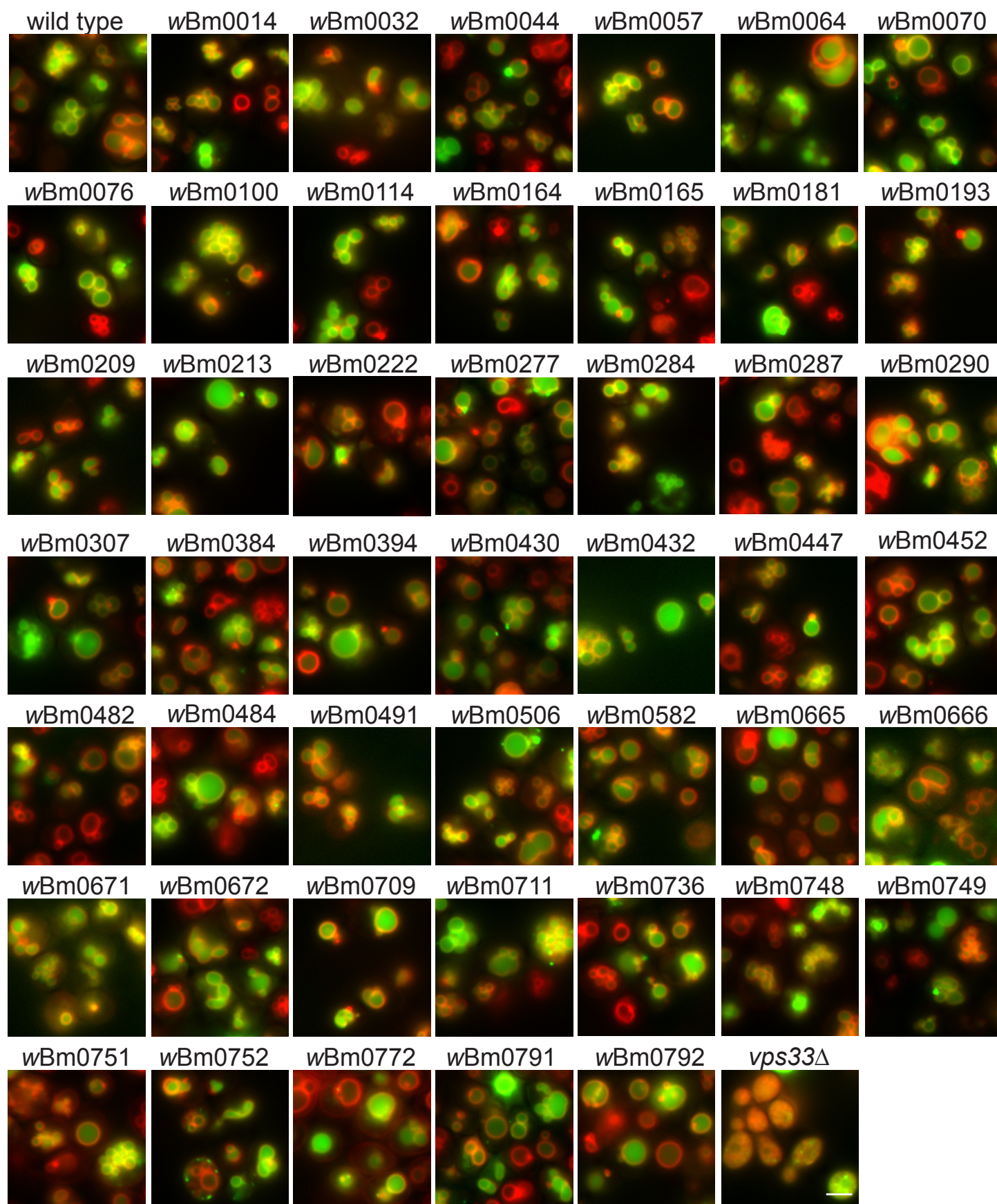

Supplement: S4 Fig — BY4742 yeast strains modified with GEV for β-estradiol-dependent induction of GAL promoters (Materials and Methods), and harboring pGO45 GFP-CPS plasmid in addition to pYES2/NT A control plasmid or pYES2/NT A harboring an individual wBm open reading frame were grown to saturation in CSM-lysine-uracil medium. Cells were subcultured to CSM-lysine-uracil with 1 μM β-estradiol and grown for 6 h at 30°C. Cells were stained with 10 μM FM46-4 for 20 minutes at 30°C, chased for 1.5 h in CSM-lysine-uracil medium at 30°C, then visualized. Representative crops from two independent experiments are shown; bar = 3 μ. (PDF) [file pone.0204736.s005.pdf]

# Figure S5

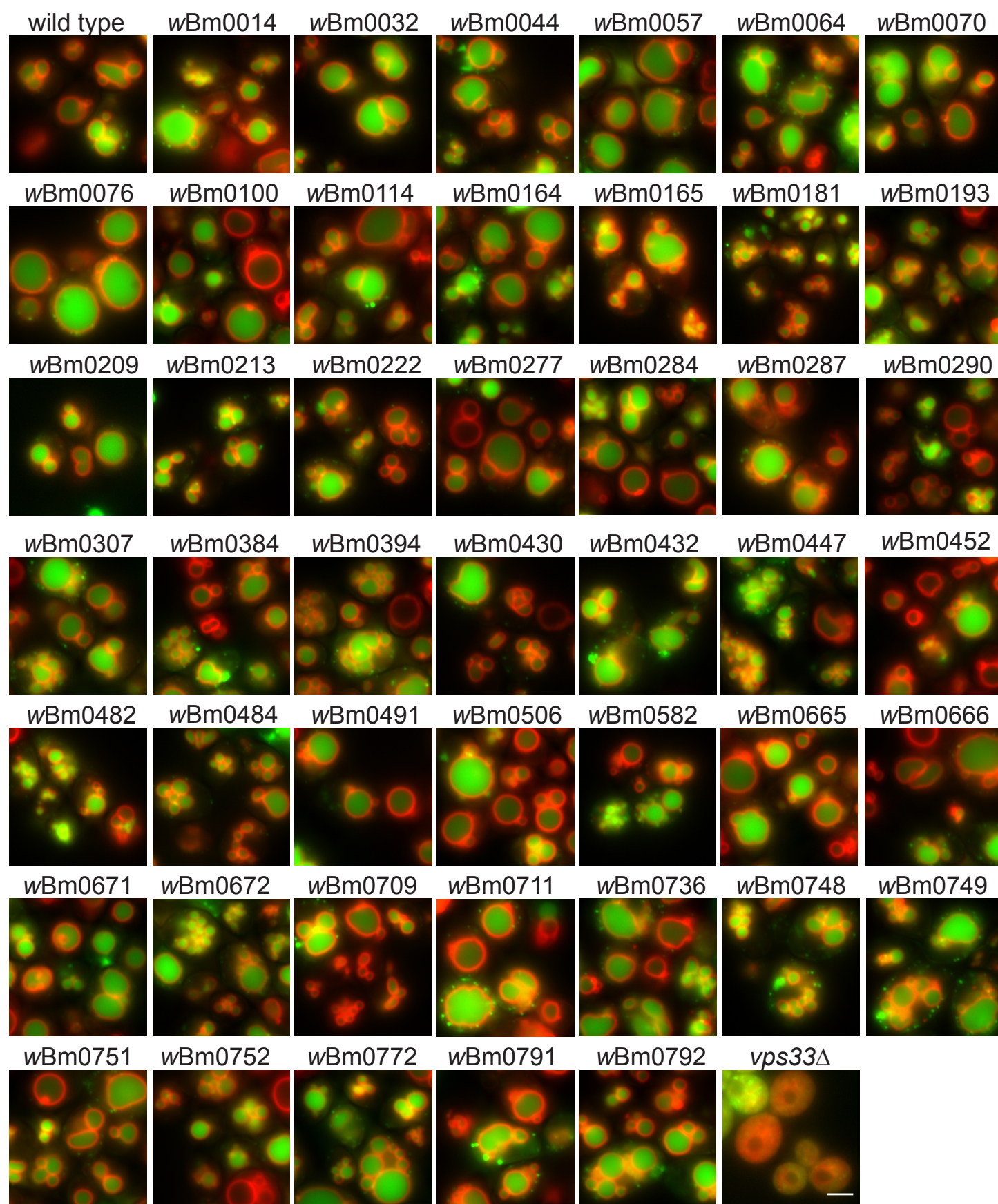

Supplement: S5 Fig — BY4742 yeast strains modified with GEV for β-estradiol-dependent induction of GAL promoters (Materials and Methods) and harboring the Sna3-GFP plasmid in addition to pYES2/NT A control plasmid, or pYES2/NT A harboring an individual wBm open reading frame, were grown to saturation in CSM-lysine-uracil medium. Cells were subcultured to CSM-lysine-uracil with 1 μM β-estradiol and grown for 6 h at 30°C. Cells were stained with 10 μM FM46-4 for 20 minutes at 30°C, chased for 1.5 h in CSM-lysine-uracil medium at 30°C, then visualized. Representative images from two independent experiments are shown; bar = 3 μ. (PDF) [file pone.0204736.s006.pdf]

# Figure S6

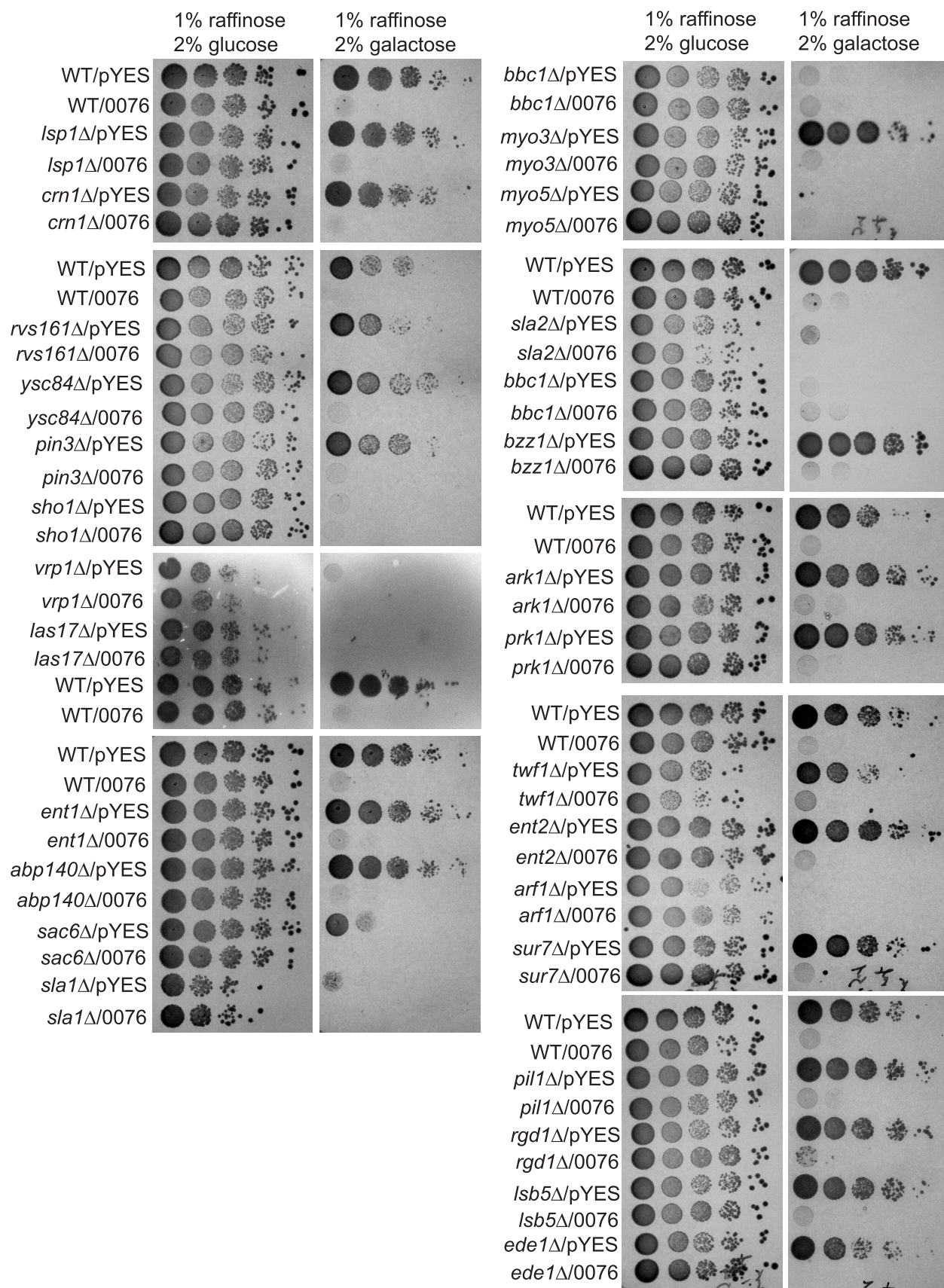

Supplement: S6 Fig — BY4742 yeast strains deleted for the indicated gene and harboring either pYES2/NT A or pYES2/NT A wBm0076 (0076) were grown overnight in CSM medium lacking uracil. Cultures were diluted to an OD600 = 1.0 in sterile 0.9% NaCl, then spotted in 10-fold dilutions on plates containing 1% raffinose and either 2% glucose or 2% galactose to induce WBM0076 expression. Plates were incubated for 72 h at 30°C and imaged; results are representative of three independent experiments. (PDF) [file pone.0204736.s007.pdf]
